# Transcriptomic analysis of phage-mammalian cell interaction reveals diverse phage immunobarcodes

**DOI:** 10.1016/j.isci.2025.113513

**Published:** 2025-09-05

**Authors:** Caroline Munini Muema, Belinda Kibii, Mingyue Zhong, Xinfeng Li, Paulina Miernikiewicz, Heng Xue, Yiyao Wang, Raphael Nyaruaba, Krystyna Dąbrowska, Hongping Wei, Hang Yang

**Affiliations:** 1 State Key Laboratory of Virology and Biosafety, Wuhan Institute of Virology, Chinese Academy of Sciences, Wuhan 430071, China; 2 University of Chinese Academy of Sciences, Beijing 100049, China; 3 Institute of Immunology and Experimental Therapy, Polish Academy of Sciences, Weigla 12, 53-114 Wrocław, Poland; 4 Faculty of Medicine, Wroclaw University of Science and Technology, 50-370 Wrocław, Poland; 5 Hubei Jiangxia Laboratory, Wuhan 430200, China

**Keywords:** Molecular biology, Immunology, Microbiology, Transcriptomics

## Abstract

The phage-mammalian cell interactome is an emerging research frontier essential for uncovering the diverse impacts phages may have on humans. Here, we investigate the crosstalk of six distinct *Acinetobacter baumannii* phages with A549 epithelial cells and RAW264.7 macrophages. Our findings indicate that phage internalization rate varies depending on the phage type and the cell line. Notably, podovirus phage demonstrates the highest rate of internalization, while myovirus phage exhibits the lowest. Internalized phages maintain significant activity against intracellular bacteria. RNA sequencing revealed that phage-treated A549 cells display anti-inflammatory transcriptional signatures (immunobarcodes). Conversely, macrophages treated with siphovirus phage CK02 or podovirus phage CK21 demonstrate an anti-inflammatory profile, while those treated with myovirus phage CK12 maintain a pro-inflammatory response. Moreover, both phage-treated cells exhibit the downregulation of cellular reproduction and proliferation pathways, pointing to underexplored dimensions of phage-mammalian cell interactions. Altogether, our findings highlight the complexity of phage effects on mammalian cells.

## Highlights

• Phages trigger distinct transcriptomic immunobarcodes in mammalian cells
• Phage-primed immunobarcodes are both cell-type- and phage-dependent
• Some immunobarcodes are shared across all phages, and others are unique to specific phage
• Phages remain active inside cells for 24 h while trapping intracellular *A. baumannii*

## Graphical abstract

**Figure:**
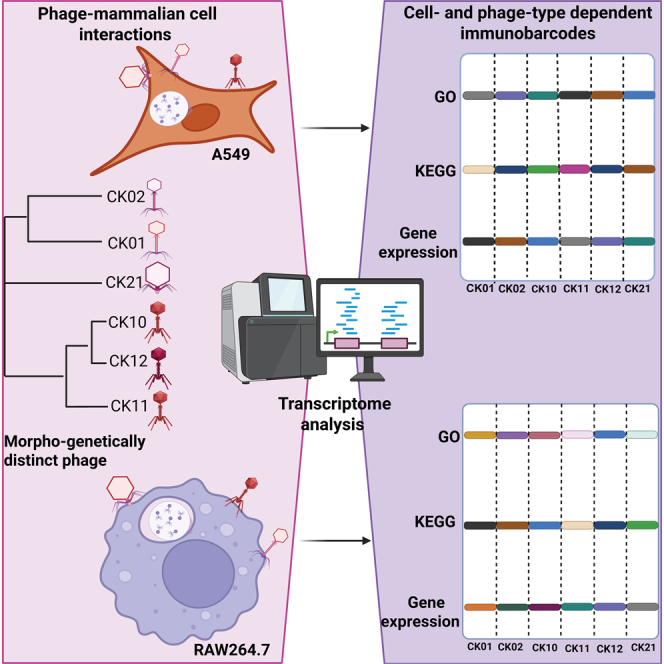

## Introduction

Research encompassing microbiota-host interactions and their correlation to health and disease has garnered increased interest over the years. Consequently, a wealth of information, particularly on bacterial-host interactions, has been acquired, while the phage-mammalian host dynamics are understudied. Phages, the most abundant and ubiquitous microorganism, are found in diverse environments including the human body, where they interact with cells across various organs.^1^^,^^2^^,^^3^^,^^4^^,^^5^

Phage interactions with human cells may encompass several processes, including cell surface interactions, uptake, internalization, and phage intracellular processing. Initial interactions occur randomly along the apical surface of the cells, where phage can bind to mammalian-associated glycans such as mucin glycoproteins located in the outer glycocalyx.^6^^,^^7^ Additionally, phage can interact directly with membrane-bound receptors such as T cell receptors, β3 integrin receptors,^8^ and sialic acid receptors^9^ and non-specifically with other surface structures followed by internalization.^10^

Phage internalization occurs via four pathways: primarily via macropinocytosis or phagocytosis, and to some extent via caveolar or clathrin-mediated endocytosis, depending on receptor availability on mammalian cells and phage size.^11^ The mechanism of phage internalization determines the mode of intracellular trafficking, which influences the eventual fate of the phage. Following uptake, phages bound in endocytic vesicles can persist in cells for a prolonged period.^9^^,^^12^ The phages can either undergo degradation^13^^,^^14^ within the vesicles or, as has been reported, some phages may escape and enter the cell cytoplasm.^15^^,^^16^ Intracellular phages may stimulate a range of intracellular pathways, including the stimulation of immune responses. Endosome-bound phages and their components may interact with endosomal Toll-like receptors, including TLR3, TLR7, and TLR9, triggering immune responses. Outside the endosome, phage and phage components may interact with cytosolic immune receptors such as RIG-I-like and NOD-like receptors which sense viral RNA and bacterial products, respectively. The activation of these intracellular receptors may trigger a cascade of immune responses including both anti-inflammatory and pro-inflammatory signaling pathways that could have diverse effects on the disease state of the host. A recent study reported that *Escherichia, Bacteroides*, and *Lactobacillus* phages and phage nucleic-acids interacted with TLR9 and stimulated an interferon-γ (IFN-γ)-mediated immune response.^17^ Similarly, Pf4 phage activated type I interferon and inhibited TNF production via interactions with TLR3, which prevented the clearance of host bacterial infection.^16^ Additionally, *S. aureus* phage exhibited anti-inflammatory effects in bovine mammary cells by suppressing inflammation induced by LPS.^18^ Furthermore, the oral administration of a phage cocktail caused the reduction of IL-1β, TNFα, and IL-6, attenuating inflammation in weaning pigs.^19^ These findings highlight the dual pro-inflammatory and anti-inflammatory roles of phages, which remain a topic of debate, emphasizing the need for more comprehensive research to unravel the mechanisms behind these interactions. Additionally, the lack of standardized methods for studying phage-mammalian cell interactions presents a significant challenge in developing comprehensive assays and drawing definitive conclusions. Furthermore, most studies on these interactions have utilized a limited number of phages, making it difficult to draw general conclusions. These limitations, coupled with the existing knowledge gaps, underscore the need for further studies on phage-mammalian host interactions to enhance the effectiveness of phage therapy.

In this context, the current study explores the interactions of six *A. baumannii* phages with mammalian epithelial and immune cells, using *in vitro* assays and transcriptomic methods, with a particular emphasis on their effects on mammalian cells. Herein, we introduce the concept of phage immunobarcode to describe the unique transcriptional signatures triggered by individual phages in different cell types. These barcodes represent immunomodulatory profiles specific to individual phages across different mammalian cell types, shaped by their genomic composition, structure, and interaction with mammalian cells. The phage immunobarcode framework thus provides a valuable lens to classify and compare the immunomodulatory potential of different phages, offering insights relevant for the development of phage-based therapeutics.

## Results

### Phages display genetic, lytic, and morphological diversity and show no cytotoxic effects on macrophage cells

**Figure 1:**
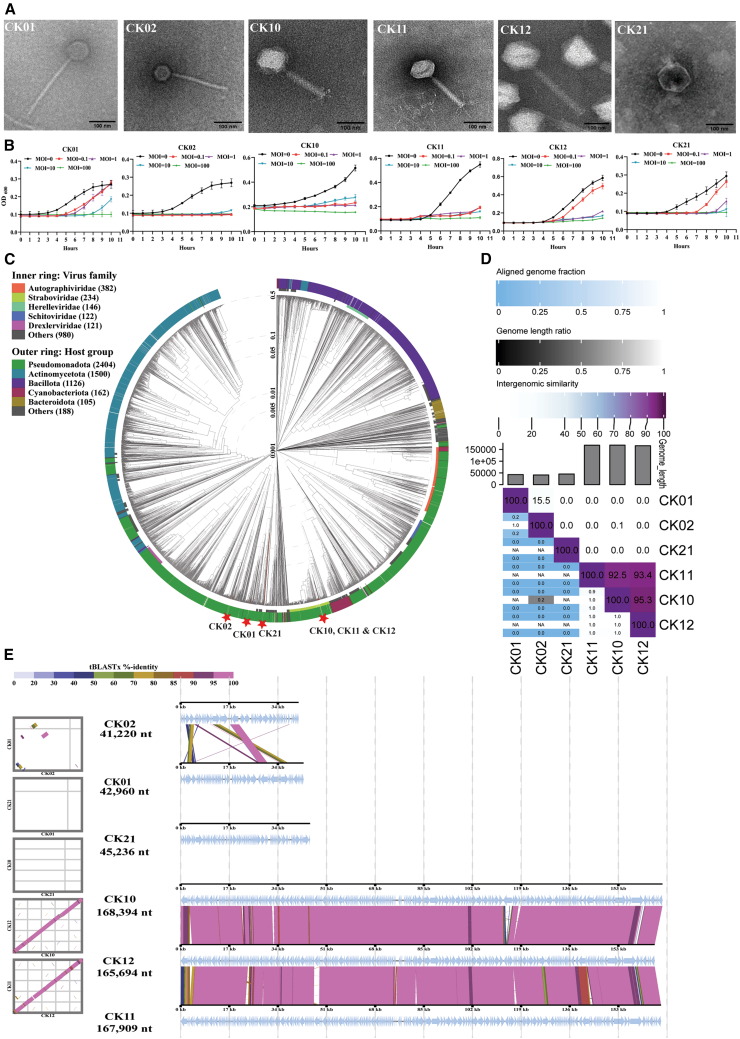
*A. baumannii* phages display extensive morphological and genetic diversity (A) Transmission electron micrographs. Scale bar represents 100 nm. (B) *In vitro* antibacterial activity of *A. baumannii* phage CK01, CK02, CK10, CK11, CK12, and CK21 against their designated bacterial hosts (*A. baumannii* WHG40042, WHG40042, WHG40090, WHG40149 LB8R, WHG40149 LB8R, and WHG40004, respectively) for 10 h at different MOIs. (C) Viral phylogenetic tree (ViPTree) of *A. baumannii* phage genomes (red asterisk) and other 3589 prokaryotic dsDNA phage genomes based on whole genome-based similarities calculated using tBLASTx. The colored rings represent the virus families (inner ring) and host groups (outer ring). (D) Heatmap showing percentage intergenomic sequence similarities (upper right half) and alignment genome fraction and genome length ratios (lower left half) for the six *A. baumannii* phages computed using VIRIDIC. (E) Whole genome comparative analysis between the six-phages based on tBLASTx. The shading below each genome indicates sequence similarities between the genomes, with different colors representing the levels of similarity according to the legend at the top left.

Six virulent phages designated as vB_AbaS_CK01, vB_AbaS_CK02, vB_AbaM_CK10, vB_AbaM_CK11, vB_AbaM_CK12,and vB_AbaP_CK21 (referred to as CK01, CK02, CK10, CK11, CK12, and CK21 from here on) were successfully isolated from wastewater and sewage samples in Wuhan using 4 clinical *A. baumannii* as indicator hosts. The transmission electron micrographs showed that all phages belong to the class *Caudoviricetes*^20^ with distinct morphotypes. Specifically, phage CK01 and CK02 exhibited the siphovirus morphology, with isometric capsids of about 78.73 and 52.19 nm in diameter and long non-contractile tails of about 269 and 211 nm long, and 12.86 and 13.48 nm wide, respectively. Phage CK10, CK11, and CK12 classified into the myovirus morphology, showing icosahedral-isometric heads with diameters of 111.95, 79.07, and 145.61 nm and contractile tails of 174.61, 164.71, and 284.8 nm long and 26.71, 16.51, and 32.88 nm wide, respectively, while phage CK21 exhibited a podovirus morphology with an icosahedral head of 103.46 nm and a short tail of 22.6 nm long and 11.68 nm wide (Figure 1A). *In vitro* co-culture assay showed that each phage displays an MOI-dependent antibacterial activity, and robust growth inhibition was observed in phage CK02 and CK11, which suppressed bacterial growth at all MOIs applied (Figure 1B). Whole genome sequencing analysis revealed that these phages contained linear dsDNA of 42,960 bp, 41,220 bp, 168,394 bp 167,909 bp, 165,694 bp, 45,236 bp and a GC content of 45.38%, 46.97% 36.43%, 39.43%, 36.35%, and 37.92% for CK01, CK02, CK10, CK11, CK12, and CK21, respectively (Figure S1; Table S1). Phylogenetic analysis using the Viral Phylogenetic Tree (ViPTree)^21^ tool revealed that phages CK01, CK02, and CK21 were taxonomically distinct from one another, while phages CK10, CK11, and CK12 were more taxonomically close (Figure 1C). To further investigate the genetic variability and evolutionary relationships among these phages, a genome-wide phylogenetic tree was constructed using VICTOR^22^ and results showed that these phages formed two major clades. Specifically, phage CK01 and CK02 formed the first clade, shared a common ancestor and clustered within the same family and genus but were different at the species level (Figure S2). Phage CK10, CK11, and CK12 were classified within the same family and genus, but CK10 and CK12 were more closely related. In contrast, phage CK21, despite being part of the second clade, was more distantly related, belonging to its own family, genus, and species (Figure S2). These findings were corroborated by intergenomic distance calculations performed using VIRIDIC, where the highest genomic similarity (95.3%) was observed between phage CK10 and CK12 (Figure 1D). While phage CK01 and CK02 showed 15.5% similarity, and phage CK02 and CK10 shared only 0.1% similarity (Figure 1D). Comparative genomic analysis further elucidated that phage CK10, CK11, and CK12 shared extensive genomic homology, while phage CK01 and CK02 exhibited limited regions of genomic similarity (Figure 1E). Phage CK21, however, displayed a highly unique genome with no significant similarity to any of the other phages (Figure 1E). These results collectively highlight the genetic diversity and distinct evolutionary trajectories of these *A. baumannii* phages. To test their cytotoxicity against mammalian cells, each phage was purified by subjecting phage lysates to PEG concentration, filtration, CsCl density gradient ultracentrifugation, and finally passing through an endotoxin removal resin (Figure S3). Then, the cytotoxicity of each purified phage toward the RAW264.7 murine macrophages was assessed using the CCK-8 assay. Results showed that all phages at a final concentration of 10^7^ PFU/well had no significant effects on the viability of RAW264.7 cells after a 24-h co-culture. In contrast, challenge with commercial *E. coli*-originated LPS resulted in a significant reduction (*p* = 0.038) in cell viability (Figure S4). The cytotoxic effects of phage and LPS on epithelial cells were not evaluated, as macrophages are more sensitive, and the results obtained from this experiment are deemed sufficiently conclusive.

### Phages exhibit differential rates of uptake, survival, and bacterial killing capacity in mammalian cells

**Figure 2:**
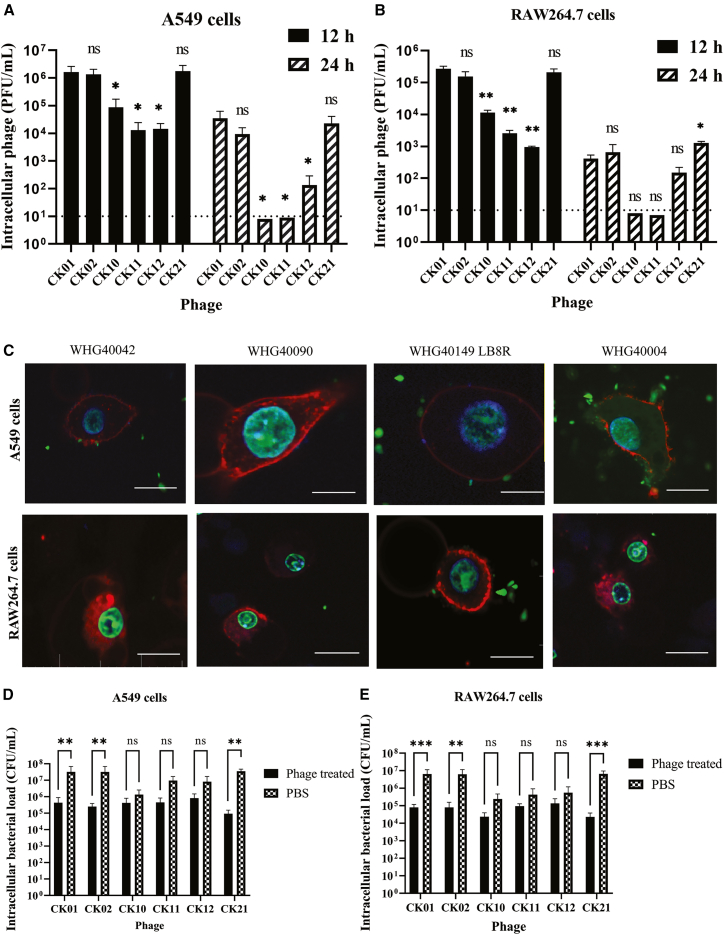
Internalization, survival, and antibacterial activity of phages in A549 and RAW264.7 cell lines (A and B) Bar graphs showing the number of internalized phage particles after 12 h, and the number of viable-surviving intracellular phage particles after 24-h in A549 (A) and RAW264.7 cells (B). The initial concentration of phage added to the cells was 10^9^ PFU/well. Statistical significance for each treatment is presented relative to the CK01-treated group. (C) Fluorescence microscopy images show intracellular *A*. *baumannii* strain WHG40042, WHG40090, WHG40149 LB8R, and WHG40004 in A549 and RAW264.7 cells. The cell membranes are stained with Dil (red), the nuclei are stained with DAPI (blue), and the bacteria are stained with SYBR green dye (green). Scale bars, 5 μm. (D and E) Intracellular bacterial loads in A549 (D) and RAW264.7 cells (E) infected with *A. baumannii* host strains and treated with respective phage for 8 h. Phage and bacterial quantification results are presented as mean ± SD of three independent experiments. ns: not significant; ∗: *p* ≤ 0.05; ∗∗: *p* ≤ 0.01; ∗∗∗: *p* ≤ 0.001 by one-way ANOVA. Dashed line represents the limit of detection.

Uptake rate is an important measure since it sheds light on the number of phage particles that enter cells and could influence the outcome of phage-cell interactions. We therefore determined the rate at which phage could be internalized by A549 and RAW264.7 cell lines for 12 h. Our data showed that most phages were readily internalized, with phage CK01, CK02, and CK21 exhibiting higher rates of internalization compared to phage CK10, CK11, and CK12 (Figures 2A and 2B).

To determine the survival of internalized phage, we challenged A549 and RAW264.7 cells with phage, allowed uptake and internalization for 12 h, followed by 24 h of further incubation with media only. After 24 h, intracellular phages were recovered from the cells and active phages quantified. Results showed that phage survival was highly variable between them, with phage CK01, CK02, and CK21 having significant amounts of viable phage present inside the cells despite the long exposure time (Figures 2A and 2B). The highest titer loss was observed in phage CK10 and CK11, where very few particles were detectable after the 24-h chase period in both cell lines. The titer loss was more significant in RAW264.7 cells compared with A549 cells, probably due to rapid phage degradation in the phagosome. Similarly, a lower number of phages was noted in macrophages at 12 h compared with the epithelial cell line.

Recent studies have reported the ability of *A*. *baumannii* to be internalized by mammalian cells, suggesting a potential intracellular niche that may contribute to immune evasion and pathogenicity. To verify whether the clinical strains used in our study can similarly enter and persist within host cells, we performed a co-culture experiment combined with confocal imaging to directly observe bacterial internalization and persistence in mammalian cells. Results showed that *A*. *baumannii* strains tested could be internalized by both A549 and RAW264.7 cell lines (Figures 2C and S5), therefore, we further evaluated the intracellular killing capacity of phages. To this end, A549 and RAW264.7 cells were infected with *A. baumannii* strains that are susceptible to polymyxin B with an equal MIC value of 1.25 μg/mL (Table S1). After removing extracellular bacteria by polymyxin B as described,^23^ cells were then treated with the respective phage, and intracellular surviving bacteria were quantified. Results demonstrated that phage treatment significantly reduced the intracellular bacterial burden. Phage CK01, CK02, and CK21, which could persist for longer periods inside the cells, caused a notable reduction in intracellular bacteria, achieving a 1.87, 2.1, and 2.3 logs reduction in A549 cells (Figure 2D), and 1.94, 1.90, and 2.5 log reduction in RAW264.7 cells (Figure 2E), respectively. Conversely, owing to their poor intracellular survival rate, phage CK10, CK11, and CK12 exhibited negligible intracellular bacterial killing capacity in both cell lines. Moreover, the enhanced bacterial killing observed in macrophage cells may be partly attributed to their ability to kill bacteria directly and their phagocytic uptake of phage, which facilitates the delivery of phage to intracellular bacteria (Figure 2E).

### Differential gene expression in A549 and RAW264.7 cells exposed to phage

To identify the changes in cellular transcripts in response to phage, A549 cells were stimulated with 1 × 10^7^ PFU/well purified phage at a final phage to cell MOI of 10, or an equivalent volume of PBS for 12 h. After total RNA isolation and high throughput sequencing, a total of 21 independent libraries were constructed with an average of >42 million reads for each sample after cleaning and quality checks. The clean reads were aligned to the GRCh37 reference human genome using the TopHat2 software (v2.1.1).^24^ Of the total number of reads, approximately >95% could be mapped onto the human genome (Table S2). Generally, gene expression levels among the different biological replicates of the samples were highly correlated (*r* ≥ 0.95; Figure S6A), indicating that the transcriptome data were suitable and reliable for analysis of differential gene expression.

For RAW264.7 cells, we selected double purified phage CK02, CK12, and CK21 (Figure S3; Table S1), which belong to varied taxonomic classification clades and represent the siphovirus, myovirus, and podovirus morphology, taking 0.1 EU/mL of commercial LPS as a background control and PBS as a negative control. The cells were then incubated with 1 × 10^7^ PFU/well purified phage at a final phage to cell MOI of 10 for 6 and 24 h to capture early and long-time exposure reactions of cells to phage challenge. This purification strategy, experimental schedule, and control parameters were specifically applied for RAW264.7 cells, as these immune cells exhibit heightened sensitivity to immunological stimuli. Their innate immune responsiveness leads to more dynamic changes in intracellular signaling pathways compared to epithelial cells, necessitating careful experimental design to capture phage-specific immunological effects accurately.

Thirty independent libraries were constructed with >38 million clean reads each. The clean reads were then compared to the reference genome using the HISAT2 software (v2.2.1).^25^ Approximately >86% of the clean reads could be mapped to the mouse genome (GRCm38) (Table S3). Pearson correlation coefficient across different treatments was ≥0.94 (Figure S6B).

**Figure 3:**
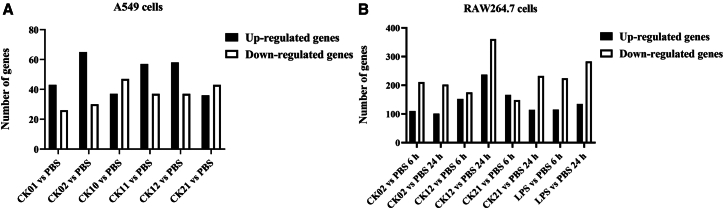
Differential gene expression profiles in A549 and RAW264.7 cells after phage challenge Bar graphs representing the number of differentially expressed genes per sample after stimulation with phages, compared with PBS-treated A549 cells for 12 h (A) and RAW264.7 cells for 6 and 24 h (B). Criteria for determining expression: log 2-fold change ≥1, significance *p-value* of ≤0.05. Each bar represents the number of genes up-regulated or down-regulated following the stimulation of cells with phage.

Transcriptome analyses of phage-stimulated versus unstimulated A549 cells (PBS-treated) revealed a total of 296 up-regulated genes and 220 downregulated genes. Phage CK02 and CK11 induced the strongest transcriptional response with 95 differentially expressed genes (DEGs) each, while CK01 induced the least cellular response with only 69 DEGs (Figure 3A; Table S4). In macrophages, phage stimulation induced a time-dependent increase in transcriptional changes, where 428 and 452 genes were upregulated while 534 and 795 were downregulated after 6 and 24 h of phage stimulation, respectively. This clearly shows a strong and dynamic response in macrophages compared to epithelial cells even as early as 6 h post-stimulation; of note, different cell lines respond differentially to phages. Furthermore, the 24-h challenge led to a greater increase in the number of DEGs, showing that responses of macrophages are also time-dependent (Figure 3B). All DEGs for A549 and RAW264.7 cells are listed in Excel Supplementary files Tables S4, S5, S6, S7, and S8.

### Common and unique cellular response profiles observed after phage challenge

**Figure 4:**
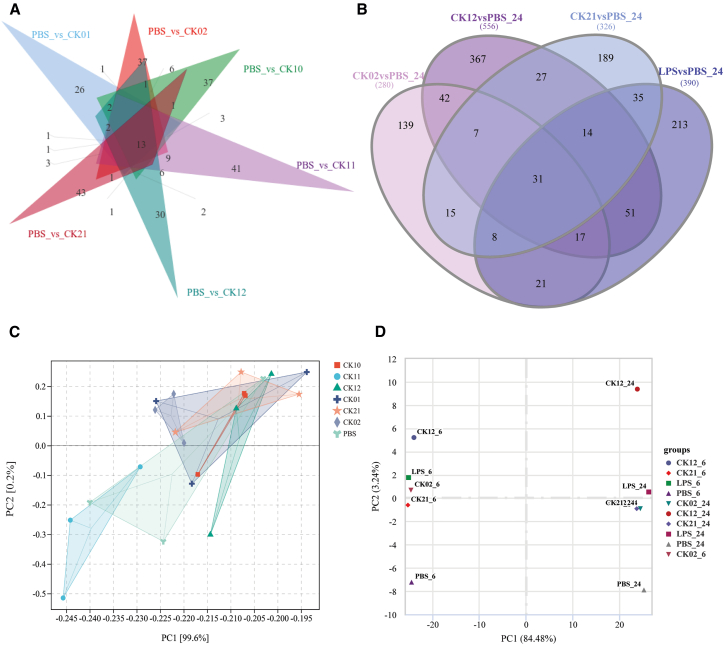
Unique and common gene expression profiles across phage-treated samples The Venn diagrams show the number of genes uniquely expressed and the gene-overlap relationships among varied phage treatments in A549 cells (A) and RAW264.7 cells (B). (C and D) PCA analysis of DEGs of different phage and control treatments in A549 cells (C) and RAW264.7 cells (D).

To assess gene expression patterns in response to phage challenge, we generated co-expression Venn diagrams to identify genes uniquely or commonly expressed across different phage treatments. We observed that each phage generated a distinct gene expression profile, where 26, 37, 37, 41, 30, and 43 genes were uniquely expressed in A549 cells treated with phage CK01, CK02, CK10, CK11, CK12, and CK21, respectively (Figure 4A). In RAW264.7 cells, 139, 367, 189, and 213 genes were unique to phage CK02, CK12, CK21, and LPS treatment, respectively (Figure 4B). These results show that phages possess distinct properties that exert unique effects on mammalian cells. Despite the phage-specific gene expression profiles, we identified 13 and 7 genes commonly expressed across all phage-treated groups in A549 and RAW264.7 cells, respectively (Figures 4A and 4B). Among the 13 genes in A549 cells, 9 were up-regulated (PER1, CYP1B1, TIPARP, FKBP5, KLF9, AHRR, CYP1A1, MCF2L, and BBS1) and 4 were down-regulated (MYOCD, IL11, SOX9, and ID4) following phage exposure (Table S9). In RAW264.7 cells, 2 (4933426B08Rik, and F2) were up-regulated, while 5 (Gm50270, AC122821.1, TNXB, Gm43164, and Gm38346) were down-regulated (Table S10). These findings suggest that mammalian cells share certain conserved cellular responses to phage challenge.

Principal component analysis (PCA) of DEGs in A549 cells revealed that phage-treated samples predominantly clustered on the upper right quadrant, with the exception of CK11 and PBS, which localized toward the bottom left quadrant and center, respectively (Figure 4C). In contrast, PCA analysis of the DEGs in RAW264.7 cells demonstrated distinct differences in gene expression among the different phages, and between short (6 h) and prolonged (24 h) phage exposure. Notably, the PBS control group was distinctly separated from the phage-treated groups (Figure 4D). These results further solidify the distinct effects of phage on mammalian cells and demonstrate the influence of treatment duration on cellular responses.

### Phages induce anti-inflammatory signatures in A549 cells

**Figure 5:**
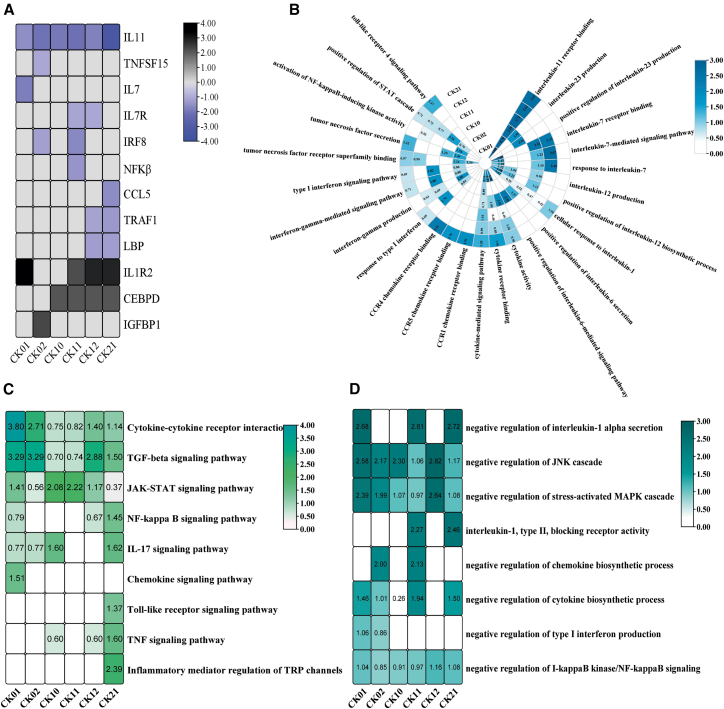
Anti-inflammatory transcripts in A549 cells challenged with phage (A) Heat maps representing repressed expression (blue) of inflammatory genes and upregulation (black) of anti-inflammatory genes after phage treatment. Gene expression is shown as log_2_(fold change). (B) GO enrichment analysis of downregulated genes shows the enrichment of inflammatory terms. Each term is represented as -log_10_(*p*-value). (C) KEGG enrichment analysis of downregulated genes, indicating the enrichment of pro-inflammatory signaling pathways. Enrichment of each pathway is represented as -log_10_(*p*-value). (D) GO enrichment analysis of upregulated genes indicating the negative regulation of inflammatory pathways in A549 cells after treatment with phage for 12 h. Enrichment of each pathway is shown as -log_10_(*p*-value).

Since various immune response components have been implicated in cellular responses to phage, it was of great interest to survey our transcriptome results for any alterations in immune-related genes. We therefore examined the expression levels of immune, cytokine, and chemokine genes. Intriguingly, RNA-seq analysis revealed that purified phage did not activate immune-related genes after 12 h of stimulation in A549 cells. Alternatively, the results revealed a suppression of several pro-inflammatory cytokines, chemokines, and inflammatory genes, including a notable downregulation of IL11 by all phages. In addition, phage CK01 caused the downregulation of IL7; phage CK02 caused the downregulation of IRF8 and TNFSF15; phage CK11 caused the downregulation of NF-κB, IRF8, and IL7R; phage CK12 caused the downregulation of LBP, TRAF1, and IL7R; while phage CK21 led to the downregulation of LBP, TRAF1, and CCL5 (Figure 5A). Notably, phage also caused the upregulation of anti-inflammatory cytokines and genes, including IL1R2, CEBPD, and IGFBP1 (Figure 5A; Table S4).

GO enrichment analysis of downregulated genes revealed significant enrichment of processes involved in the production, biosynthesis, activity, and regulation of inflammatory chemokine and cytokine signaling pathways. These include key immunological mediators such as interleukin 1, 6, 7, 8, 11, 12, 23, as well as IFN-γ, CCR1, CCR5, and CCR4, all of which are potent inflammatory cytokines. Additionally, downregulated genes enriched pro-inflammatory pathways, including the IFN-γ, TNF, NF-κB, JAK-STAT, JNK, and Toll-like receptor signaling pathways (Figure 5B).

Moreover, KEGG enrichment analysis of downregulated genes indicated significant downregulation of pathways involved in pro-inflammatory responses, including cytokine-cytokine receptor interaction, TGF, JAK-STAT, IL17, NF-κB, TNF, and TLR signaling pathways (Figure 5C). Furthermore, pattern recognition receptor pathways such as the NOD-like receptor pathway and cytosolic DNA sensing pathway, which detect pathogen-associated molecular patterns and activate cytokine secretion, were also enriched by downregulated genes, further strengthening the anti-inflammatory activity of the phage (Figure 5C).

Complementary GO enrichment analysis of upregulated genes in A549 cells revealed enrichment of pathways that repress inflammation, including the negative regulation of interleukin-1-alpha secretion and production, the negative regulation of JNK and MAPK cascade, receptor blocking activity against type II interleukin 1, and the regulation of cytokine production involved in inflammatory response (Figure 5D).

These findings indicate that the anti-inflammatory effects of phage can be achieved both by the induction of anti-inflammatory genes and the repression of pro-inflammatory genes and pathways, thereby highlighting their potential as inflammatory modulators in epithelial cells.

### Phages induce variable immunomodulatory responses in RAW264.7 cells

Contrary to the anti-inflammatory profile observed in A549 cells, both phage and LPS treatment led to the induction of pro-inflammatory genes, cytokines, transcriptional regulators, and surface markers, including CXCL2, CXCL10, TNF, IL6, CCL5, IL10, IL2, TNF, and IFNB1, in the macrophage cells (Supplementary Excel Tables S5, S6, S7, and S8).

**Figure 6:**
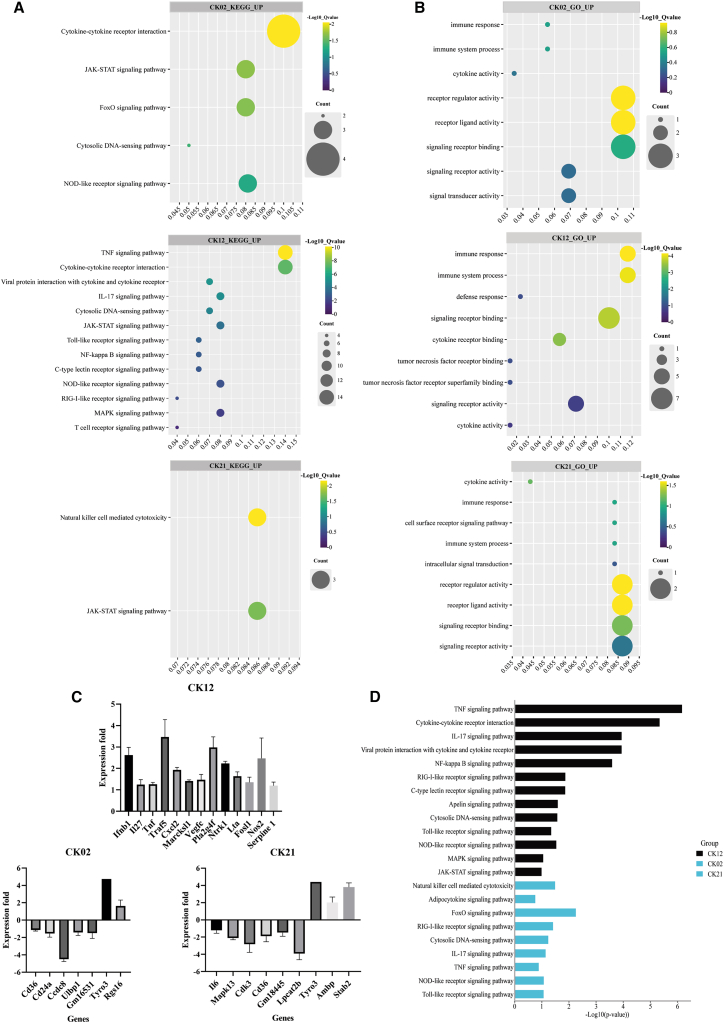
Phages activate cellular immune-response transcripts in RAW264.7 cells Bubble plots representing (A) KEGG and (B) GO enrichment analysis of upregulated genes in RAW264.7 cells after 24 h of phage treatment in comparison to PBS treatment. The color represents the *p*-value range while the dot size represents the number of upregulated genes mapped to the indicated pathway. The figures show selected pro-inflammatory pathways, and the enrichment of each pathway is represented as -log_10_(*p*-value). (C) Bar graphs showing expression of upregulated pro-inflammatory genes after treatment with phage CK12, and expression of downregulated pro-inflammatory and up-regulated anti-inflammatory genes after the treatment of RAW264.7 cells with phage CK02 and CK21, in transcriptomic comparison of phage treated vs. LPS treated cells. The expression of each gene is expressed as log_2_(fold change). Error bars depict the standard error of the mean. (D) Bar graphs showing KEGG enrichment analysis of upregulated genes after macrophage treatment with phage CK12 and downregulated genes after treatment with phage CK02 and CK21 in transcriptomic comparison of phage treated vs. LPS treated cells. The enrichment of each pathway is represented as -log_10_(*p*-value). Color coding distinguishes upregulated (black) and downregulated (blue) gene-associated pathways.

KEGG enrichment analysis showed that upregulated genes enriched key inflammatory pathways, including the NF-κB, TNF, IL17, JAK-STAT, MAPK, and FOXO signalling pathways, cytokine-cytokine receptor interaction, and viral protein interaction with cytokine and cytokine receptor (Figure 6A). Additionally, upregulated genes also led to the activation of pattern recognition receptor pathways, including RIG-I-like receptor signaling pathway, Toll-like receptor signaling pathway, NOD-like receptor signaling pathway, and cytosolic DNA sensing pathways (Figure 6A).

GO enrichment analysis revealed that phage upregulated genes enriched terms related to the positive regulation of cytokine activity, cytokine receptor binding, defense response, immune response, tumor necrosis factor receptor binding, and signaling receptor activity (Figure 6B).

Notably, myovirus phage CK12 exhibited substantially stronger enrichment of these pathways relative to siphovirus CK02 and podovirus CK21 (Figures 6A and 6B). These pathways are integral for pro-inflammatory reactions, suggesting that phage-induced immunomodulation is mediated by a complex interplay of signaling networks. Additionally, the differential gene and pathway-enrichment patterns observed among different phages indicate that these effects are phage specific, further emphasizing the concept that each phage has a unique immunobarcode.

To determine whether the observed immune response was phage-specific and not due to residual endotoxins in phage solutions, we compared the transcriptomic profile of phage-treated cells to that of LPS (background control) stimulated cells. Notably, there was a significant shift in the immune profile of cells treated with siphovirus phage CK02 and podovirus phage CK21 to an anti-inflammatory profile. This finding shows the high sensitivity of immunological cells to residual LPS, but at the same time, also a unique immune modulation mechanism independent of residual endotoxins. The analysis revealed that macrophage challenge with phage CK02 elicited anti-inflammatory responses through the suppression of pro-inflammatory genes such as CD36, CD24a, and ULPB1, and upregulation of anti-inflammatory genes such as TYRO3 and RGS16. Treatment with phage CK21 similarly led to the downregulation of pro-inflammatory genes including IL6, CD36, and LTB4R1, and upregulation of anti-inflammatory genes such as TYRO3 and STAB2 (Figure 6C). This was accompanied by the suppression of pathways associated with inflammation, including cytokine networks such as TNF, IL17, and JAK-STAT signaling pathways. Pathogen sensing systems such as cytosolic DNA sensing, RIG-I-like receptor, and Toll-like- receptors, and signal integration systems such as the cytokine-cytokine receptor interaction, and viral protein interaction with cytokine (Figure 6D).

In contrast, the myovirus phage CK12 did not elicit an anti-inflammatory effect but instead maintained a pro-inflammatory impact. This was evidenced by the upregulation of key inflammatory genes such as TNF, IFNB1, CXCL2, and IL27 (Figure 6C). KEGG enrichment further highlighted the activation of key pro-inflammatory pathways by phage CK12, including the TNF, IL17, NF-κB, TLR, JAK-STAT, and cytokine-cytokine receptor interaction pathways (Figure 6D). These observations suggest that the observed immune responses are phage-specific and may be linked to the structural and genetic characteristics inherent to the phage.

Overall, we acknowledge the effect that residual LPS may exert on immunological cells, but these results also demonstrate that the immunomodulatory effects of phage may be cell-type and phage-type dependent. This variability suggests a unique immunobarcode may exist for each phage in different cell lines, thereby providing critical insights into the complex interactions between phage and mammalian immune system.

### Phage influence on cellular proliferation and developmental pathways

**Figure 7:**
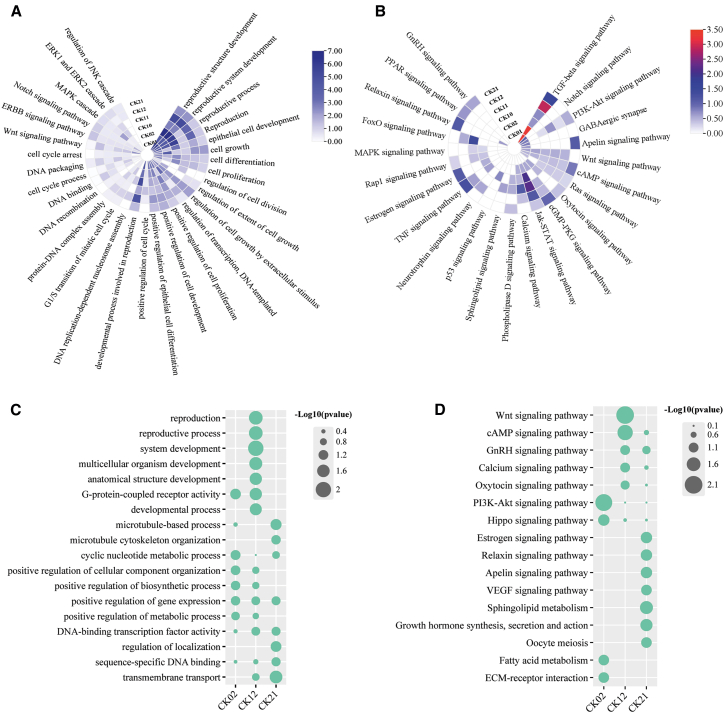
Effects of phage on cellular physiological processes Heat maps representing GO (A) and KEGG (B) enrichment analysis of downregulated genes in A549 cells and GO (C) and KEGG (D) enrichment analysis of downregulated genes in RAW264.7 cells. The figures show pathways involved in cellular growth, proliferation, and development, and the enrichment of each pathway is represented as -log_10_(*p*-value).

Phage exposure significantly impacted the reproductive, proliferative, and developmental processes in both A549 and macrophage cells. For instance, GO enrichment analysis of downregulated genes in A549 cells revealed enrichment of terms associated with reproduction, including reproductive system development, cell growth, differentiation, and proliferation. Cell cycle, including DNA binding and packaging, and the G1/S phase transition of the mitotic cell cycle. Critical development signaling pathways including Erbb, Wnt, Notch signaling pathways, and survival and stress response cascades such as MAPK, ERK1/2, and JNK cascades (Figure 7A).

These results were also reflected in KEGG pathway analysis, which indicated that the downregulated genes enriched pathways involved in cell proliferation, growth, and survival, such as the TGF-beta signaling pathway, Wnt signaling pathway, Notch signaling pathway, PI3K-Akt signaling pathway, P53 signaling pathway, Apelin signaling pathway, FoxO signaling pathway, and so forth. (Figure 7B). In macrophages, GO enrichment showed that downregulated genes similarly enriched pathways involved in key physiological processes, including those involved in the regulation of metabolism, gene expression, and biosynthesis, reproduction, reproductive process, developmental process, and microtubule-based processes (Figure 7C). KEGG enrichment further confirmed the enrichment of downregulated genes in additional proliferation-associated pathways, including Wnt, cAMP, PI3K-Akt, and Hippo signaling (Figure 7D). These results were maintained in the transcriptome comparison between phage treated versus LPS treated cells, indicating that these cellular responses are possibly phage-induced.

## Discussion

Recently, phages have been discovered in sterile areas of the body characterized by anatomical and physiological barriers, raising concerns about the consequences of phage capability to bypass these barriers.^26^ These barriers include epithelial, endothelial, and mucosal cell layers, which have been demonstrated to engage in phage translocation in the body, facilitating interactions with both therapeutic and natural phage.^12^ However, little is known about phage-mammalian cell interactions, and only recently have they began to be explored. So far, it is clear that eukaryotic cells can uptake phages;^10^^,^^27^^,^^28^ however, the fate of intracellular phage and their effects on cells are not well defined. This study aimed to uncover the internalization, fate, and influence of six *A. baumannii* phages belonging to different morphology groups on epithelial (A549) and immune (RAW264.7) cells.

Our findings reveal that phage internalization rates vary significantly across different phage types and cell lines. Notably, podovirus phage CK21 exhibits the highest rate of internalization, followed by siphovirus phage CK01 and CK02, while myovirus phage CK12, CK10, and CK11 show the lowest rate of internalization. This suggests that phage size and morphology play a role in their uptake by mammalian cells. The smaller-sized phages are likely to accumulate and occupy a larger surface area of the cell membrane, increasing their abundance in endocytic pits for uptake. Importantly, phage engulfment efficiency is known to be restricted by phage size, since pits and vesicles created during the engulfment process can be insufficient to accommodate larger objects.^11^ A similar observation was also recorded in *E. coli* phages in a recent study, which showed that podovirus phage T3 had the highest uptake rate, followed by siphovirus phage lambda and then myovirus phage T4.^10^

Once internalized, phages retained viability for 24 h, however, a slight reduction in intracellular phage particles was observed in both cell lines, possibly as a result of phage extrusion from the cells or degradation in intracellular vesicles such as phagolysosomes. Previous studies have reported a loss of phage capsid outer coat when the phage was trafficked in phagolysosomes.^29^ Likewise, phage lambda particles re-isolated from the phagosome retained infectivity against their host bacteria.^30^

The ability of phages to retain extended intracellular viability offers an advantage for phage therapy, since longer phage exposure will ensure sufficient killing of intracellular bacterial pathogens that escape antibiotic and immune system clearance.^31^ One such pathogen is *A. baumannii*, which presents a facultative ability to invade, persist, and use eukaryotic cells as niches for replication.^32^^,^^33^ Furthermore, the bacteria target cellular nuclei via the outer membrane protein A (AbOmpA), which translocates to the nucleus through a monopartite nuclear localization signal (NLS).^34^ The internalization and persistence of phage CK01, CK02, and CK21 enabled them to significantly reduce intracellular *A. baumannii* hosts in both investigated cell lines. Similarly, macrophage internalized phage D29 was able to eliminate intracellular mycobacteria.^35^ These results present the prospective therapeutic application of phage for the elimination of intracellular bacteria.

Transcriptome analysis revealed a common (core) gene expression profile in mammalian cells exposed to phages, despite using phages belonging to different taxonomic groups and representing differing morphologies. The common gene expression profile could be linked to some conserved genes or proteins in phage, such as those involved in DNA replication, structural assembly, and host lysis, as demonstrated in studies on *Myoviridae* and *Podoviridae* phage.^36^^,^^37^ Importantly, unique, phage-dependent profiles were also observed, showing that phages contain distinctive immunomodulatory properties as a result of genetic variation, mutation, or evolutionary adaptation. Such phage-strain specific variations in gene expression are critical when considering the potential application of phages in therapeutic settings, as different phages may elicit distinct effects on host cells.

Several mechanisms have been proposed to explain the anti-inflammatory activity of phage, including the elimination of bacterial pathogens by phage, which reduces cellular inflammatory reactions,^38^^,^^39^ and binding of phage to LPS, rendering it unavailable to interact with cellular receptors.^40^ In our study, however, we observed a direct activation of anti-inflammatory genes such as IL1R2 and repression of inflammatory pathways such as NF-κB, TNF, TLR, and JAK-STAT signaling pathways in the epithelial cells. This suggests that phage anti-inflammatory activity can be attributed to both the direct activation of anti-inflammatory genes and suppression of pro-inflammatory genes and pathways. Interestingly, in the epithelial A549 cells, no immune activation was detected toward the phage, their proteins or nucleic acids, and pattern recognition receptors such as cytosolic DNA sensors TLR9 and cGAS-STING were not activated.

Contrary to the anti-inflammatory effect of phage in A549 cells, we observed an activation of pro-inflammatory genes and pathways in macrophages by the same phage preparations, including NF-κB signaling pathway, Toll-like receptor signaling pathway and TNF signaling pathway. This could, however, be a result of macrophage reaction to residual LPS and thus could rather be considered a failure of phage to attenuate the pro-inflammatory reaction (to LPS), possibly due to the rapid degradation of phage in macrophages. This was confirmed by performing a complementary transcriptomic comparison of phage-treated vs. LPS (background control) treated cells which revealed an attenuation of the pro-inflammatory state to an anti-inflammatory profile in cells treated with siphovirus phage CK02 and podovirus phage CK21. This was, however, not observed in cells treated with myovirus phage CK12, which maintained a pro-inflammatory immune profile even after background LPS was deducted. These results highlight the phage-specific nature of immune modulation in RAW264.7 cells. The stark differences in the immunomodulatory effects observed between siphovirus and podovirus phage versus myovirus phage suggest that structural and genetic properties may significantly influence immune responses. Beyond morphology, other factors such as variations in phage-associated proteins, endotoxin carryover, and host-derived components incorporated during replication may also contribute to these distinct immunogenic profiles. Additionally, the specific *A. baumannii* host strains used for phage propagation could influence the observed immune responses, as different bacterial strains may have unique surface components or modifications that require unique phage components to facilitate lysis. This suggests that even closely related phages can possess varying degrees of immunogenicity, potentially due to differences in capsid composition, tail fibers, or other structural elements that engage immune receptors differently. As a result, each phage may exhibit a unique immunobarcode, shaping its immunomodulatory potential. These findings highlight the potential of tailoring phage therapy to achieve targeted immune modulation, which could be particularly relevant in managing inflammatory diseases.

KEGG and GO enrichment analysis results in both A549 and RAW264.7 cells revealed that genes downregulated by phages were implicated in cell reproduction, proliferation, and developmental processes. These genes enriched pathways such as the PI3K-Akt signaling pathway, Wnt signaling pathway, cAMP signaling pathway, and ECM-receptor interaction, which are all involved in cellular processes such as cell proliferation, development, survival, and growth. To the best of our knowledge, the downregulation of genes involved in cellular proliferation, replication and reproduction following phage challenge has not been reported before, revealing new dimensions of phage-eukaryotic cell interactions. Although it is well established that phages cannot replicate within eukaryotic cells, our findings raise intriguing questions on the reason for phage influence on cellular reproduction and proliferation processes. Intriguingly, a recent study reported that T4 phage could be internalized by cells as a nutrient resource and activated the AKT pathway, which promoted cellular growth, micropinocytosis, and survival, and inhibited the CDK1 pathway, which delayed cell cycle progression and prolonged cellular growth.^41^

In sum, observations from the present study show the unique and diverse nature of phage-eukaryotic cell interactions, suggesting that the implications of these interactions are yet to be exhausted. Therefore, further research is still required to fully characterize these interactions, which may unlock potential applications of phages beyond their antibacterial properties.

### Limitations of the study

This study explores the influence of *A. baumannii* phages on mammalian cells. However, several limitations should be acknowledged. First, our study was restricted to *A*. *baumannii* phages, limiting our understanding of whether phages infecting other bacterial species elicit similar or distinct responses in mammalian cells.

Additionally, while we utilized A549 epithelial cells and RAW264.7 macrophages to model immune and epithelial interactions, these immortalized cell lines have inherent limitations, which may not fully recapitulate the responses observed in primary cells or *in vivo* systems.

Furthermore, RNA sequencing (RNA-seq) data do not directly reflect protein abundance due to post-transcriptional regulatory mechanisms. Future studies integrating proteomics and metabolomics would provide a more comprehensive understanding of the functional consequences of phage exposure.

Moreover, this study utilized commercial LPS from *E. coli* as a pro-inflammatory control, which may introduce variability in immune responses as LPS is structurally heterogeneous. And the current study cannot completely rule out the influence of residual LPS in phage preparations, although their concentrations are very low in our system. Future studies using well-characterized, strain-specific LPS preparations or alternative inflammatory stimuli could help mitigate these inconsistencies.

Lastly, our study does not delineate the precise mechanisms by which *A. baumannii* phages influence mammalian cellular processes. However, our findings establish a foundation for future research to explore the underlying molecular pathways and the broader implications of phage-mammalian cell interactions.

## Resource availability

### Lead contact

Further information and requests for resources and reagents should be directed to and by the lead contact, Hang Yang (yangh@wh.iov.cn).

### Materials availability

This study did not generate new unique reagents.

### Data and code availability

The raw RNA sequencing data reported in this work have been deposited in the Genome Sequence Archive (Genomics, Proteomics & Bioinformatics 2021) in National Genomics Data Center (Nucleic Acids Res 2022), China National Center for Bioinformation/Beijing Institute of Genomics, Chinese Academy of Sciences under accession number GSA: CRA027213 (RAW264.7 transcriptome raw data) and GSA: HRA010627 (A549 transcriptome raw data) that are publicly accessible at https://ngdc.cncb.ac.cn/gsa as of the publication date. And the phage genome data reported in this work have been deposited in the GenBase^42^ in the National Genomics Data Center,^43^ Beijing Institute of Genomics, Chinese Academy of Sciences/China National Center for Bioinformation, under accession numbers GenBase: C_AA107471.1 to C_AA107476.1, which is publicly accessible at https://ngdc.cncb.ac.cn/genbase.

Any additional information required to reanalyze the data reported in this article is available from the lead contact upon request.

## Acknowledgments

This work was supported by the 10.13039/501100001809National Natural Science Foundation of China (No. 32161133003, and 32070187 to HY), the Natural Science Foundation of Hubei Province (2024AFA093 to HY), and the National Science Centre in Poland (UMO-2021/40/Q/NZ7/00202 to KD). We thank the Institutional Center for Shared Technologies and Facilities of Wuhan Institute of Virology, CAS for their assistance in microscopy experiments.

## Author contributions

Conceptualization: M.C.M., K.D., and H.Y.; formal analysis: M.C.M., B.K., X.L., P.M., H.W., and H.Y. funding acquisition: K.D., and H.Y.; investigation: M.C.M., B.K., M.Z., H.X., Y.W., and N.R.; methodology: M.C.M., B.K., M.Z., X.L., K.D., H.W., and H.Y.; project administration: K.D., H.W., and H.Y.; resources: M.Z. and X.L.; supervision: K.D., H.W., and H.Y.; validation: M.C.M. and N.R.; writing – original: draft M.C.M. and B.K.; writing – review and editing: M.C.M., P.M., N.R., K.D., H.W., and H.Y.

## Declaration of interests

The authors declare no competing financial interests.

## STAR★Methods

### Key resources table

**Table:** 

REAGENT or RESOURCE	SOURCE	IDENTIFIER
**Bacterial and virus strains**		

*A. baumannii* strain WHG40042	Clinical isolate	N/A
*A. baumannii* strain WHG40090	Clinical isolate	N/A
*A. baumannii* strain WHG40149 LB8R	Clinical isolate	N/A
*A. baumannii* strain WHG40004	Clinical isolate	N/A
*A. baumannii* phage CK01	Huazhong agricultural university waste water	vB_AbaS_CK01
*A. baumannii* phage CK02	Huazhong agricultural university sewer	vB_AbaS_CK02
*A. baumannii* phage CK10	Tongji hospital waste water	vB_AbaM_CK10
*A. baumannii* phage CK11	Jiangxia hospital waste water	vB_AbaM_CK11
*A. baumannii* phage CK12	Pond water	vB_AbaM_CK12
*A. baumannii* phage CK21	Xiehe hospital waste water	vB_AbaP_CK21

**Biological samples**		

Sewage	Huazhong agricultural university	
Wastewater	Huazhong agricultural university	
Wastewater	Tongji hospital	
Wastewater	Jiangxia hospital	
Pond water	Pond	
Wastewater	Xiehe hospital	

**Chemicals, peptides, and recombinant proteins**		

TRIzol	Thermo Fisher Scientific	15596018
DIL	Thermo Fisher Scientific	D3911
DAPI (4’,6-diamidino-2-phenylindole dihydrochloride)	Beyotime	C1005
SYBR™ Green I	Thermo Fisher Scientific	S7563
Proteinase K	Merck Millipore	39450-01-6
DNase I	Thermo Scientific	PI90083
Fetal Bovine Serum	MilliporeSigma	F4135
Dulbecco’s Modified Eagle Medium F12 (DMEM-F12)	Fisher Scientific, GIBCO)	11320-033
Dulbecco’s Modified Eagle Medium (DMEM)	Fisher Scientific, GIBCO)	11965-092
Antibiotic-antimycotic solution (100 U/mL penicillin G, 100 mg/mL streptomycin and 0.25 μg/mL amphotericin B)	Fisher Scientific	15240062
Triton X-100	Acros Organics	327371000
LPS from *Escherichia coli* O111:B4	Sigma-Aldrich	93572-42-0
RNase A	VWR International	A2760.0100
PEG8000	Promega	V3011
Polymyxin B Sulfate	Coolaber	1405-20-5

**Critical commercial assays**		

Endotoxin removal agarose resin	YEASEN Biotechnology	20518ES10
Limulus amebocyte lysate assay	GenScript	L00350

**Deposited data**		

RNA-seq data (A549 cells)	This study	GSA: CRA023504
RNA-seq data (RAW264.7 cells)	This study	GSA: HRA010627
*A. baumannii* phage CK01 genome	This study	GenBase: C_AA107471.1
*A. baumannii* phage CK02 genome	This study	GenBase: C_AA107472.1
*A. baumannii* phage CK10 genome	This study	GenBase: C_AA107473.1
*A. baumannii* phage CK11 genome	This study	GenBase: C_AA107474.1
*A. baumannii* phage CK12 genome	This study	GenBase: C_AA107475.1
*A. baumannii* phage CK21 genome	This study	GenBase: C_AA107476.1
Human: A549 cells	ATCC	CCL-185
Mouse: RAW264.7 cells	ATCC	TIB-71

**Software and algorithms**		

ImageJ	Schneider et al.^44^	https://imagej.nih.gov/ij/
GraphPad Prism version 9.4.1	GraphPad Software, San Diego, California, USA	www.graphpad.com
Prokka 1.14.5	Seemann^45^	https://bioweb.pasteur.fr/packages/pack@prokka@1.14.5
GeneMarkS 4.28	Besemer et al.^46^	http://exon.gatech.edu/genemark/genemarks.cgi
Basic Local Alignment Search Tool	Altschul et al.^47^	https://blast.ncbi.nlm.nih.gov/Blast.cgi
VICTOR web server	Meier-Kolthoff and Göker^22^	https://ggdc.dsmz.de/victor.php
VipTree	Nishimura et al.^21^	http://www.genome.jp/viptree.
PhageScope	Wang et al.^48^	https://phagescope.deepomics.org
TopHat software	Kim et al.^27^	http://ccb.jhu.edu/software/tophat.
HISAT2 software	Kim et al.^27^	https://github.com/DaehwanKimLab/hisat2.
Cutadapt (version 1.8.1)	Martin^49^	https://cutadapt.readthedocs.io/en/stable/
Clusterprofiler	Yu et al.^50^	http://bioconductor.org/packages/release/bioc/html/clusterProfiler.html
HTSeq	Anders et al.^51^	https://pypi.python.org/pypi/HTSeq.
Adobe Illustrator	Adobe	http://www.adobe.com/products/illustrator.html

### Experimental model and study participant details

#### Bacterial strains

The four *Acinetobacter baumannii* bacterial strains were obtained from the intensive care unit of Tongji Hospital, Wuhan. The bacterial strains were grown in Lysogeny Broth (LB) at 37°C with agitation at 200 rpm and stored at -80°C in 25% glycerol.

#### Phage

Phages used in this study were isolated from wastewater from different locations in Wuhan, China, against the four *A*. *baumannii* strains as outlined in the key resources table. Phages were propagated and routinely maintained using their respective isolation host strains. Phage stocks were stored at -80°C in 25% glycerol.

#### Cell lines

The human lung adenocarcinoma epithelial cell line A549 (CCL-185) and murine macrophage cell line RAW264.7 (TIB-71) cells were obtained from the American Type Culture Collection (ATCC) and maintained in Dulbecco’s Modified Eagle Medium F12 (DMEM-F12; Fisher Scientific, 11320-033, GIBCO), and Dulbecco’s Modified Eagle Medium (DMEM; Fisher Scientific, 11965-092, GIBCO) respectively, supplemented with 10% fetal bovine serum (FBS, Sigma, F4135) and antibiotic-antimycotic solution (100 U/mL penicillin G, 100 mg/mL streptomycin and 0.25 μg/mL amphotericin B) (Fisher Scientific, 15240062) at 37°C and 5% CO_2_. The cells were stored in liquid nitrogen. The cells were routinely checked for Mycoplasma contamination and authenticated through profiling short tandem repeats. The cells have a normal karyotype.

### Method details

#### Phage isolation, purification, and endotoxin removal

Briefly, 10 mL of the wastewater and sewage samples were mixed with 10 mL phage buffer (50 mM Tris-HCl, pH 7.5, 150 mM NaCl, 10 mM MgCl_2_, and 2 mM CaCl_2_) in a sterile 100 mL conical flask. A 5 mL inoculum of host bacteria in the mid-exponential phase and 10 mL of LB were added into the flask. The mixture was incubated overnight at 37°C with 200 rpm. The culture was then centrifuged at 8000 rpm for 15 minutes and filtered through a 0.22 μm membrane. The filtrate (1 mL) was serially diluted tenfold, mixed with host bacteria at a 1:1 volume ratio and incubated at 37°C, 200 rpm for 15 minutes. The mixture was then added to 4 mL of 0.7% molten soft agar (at 50°C), mixed gently and poured on LB agar plates. The plates were then incubated at 37°C overnight to allow plaque formation. Single plaques were picked from the plates and re-cultured at least 5 times to ensure pure phage strain isolation. Phage stocks of the isolated phage were prepared using the liquid lysate method as previously described.^52^ The phage was concentrated using 1.5 M NaCl and 10% PEG 8000.^53^ The endotoxins in the phage preparations were removed in two steps, first by CsCl density gradient ultra-centrifugation.^54^ To ensure sufficient removal of CsCl salts, the phage was then subjected to dialysis using 100 kDa MWCO dialysis tubing against 3 L chilled sterile ddH_2_O in a large beaker placed on a stir plate for 30 minutes. The ddH_2_O was exchanged with 3 L pre-chilled sterile phage buffer and dialyzed for 2 hours. The buffer was exchanged one more time and the phage dialysed for 24 hours. Following 24 hours, the phage concentrate was obtained and tittered using the double agar layer method. The second step of purification was performed using the endotoxin removal agarose resin (YEASEN Biotechnology) following the manufacturer’s instructions. Endotoxin concentration was determined by the endpoint chromogenic method using the ToxinSensor™ Chromogenic LAL Endotoxin Assay Kit (GenScript Biotech Corp., USA) according to manufacturer’s instructions. Each sample was analyzed in triplicate with appropriate controls (endotoxin standards and negative controls) to ensure accuracy and rule out interference. Phage applied to macrophage cells were subjected to double purification, i.e., the CsCl ultracentrifugation and endotoxin removal resin steps were performed two times. Phage information has been summarized in Table S1.

#### Transmission electron microscopy (TEM)

To characterize the morphology of phage, 20 μL of each purified phage was placed on a carbon-coated copper grid and allowed to adsorb for 10 minutes. Adsorbed phage was then negatively stained with 2% (w/v) Phosphotungstic acid (PTA, pH 7.0) for five minutes. Stained samples were observed under the Tecnai G2 20 TWIN transmission electron microscope at 100 kV.

#### *In vitro* phage antibacterial activity

A time-kill assay was performed to determine the lytic ability of the isolated phage against their respective *A. baumannii* over a 10-hour time course. 190 μL overnight bacterial cultures at OD_600_ = 0.1 were mixed with 10 μL phage to a final multiplicity of infection (MOI) of 0.1, 1, 10, and 100 and incubated at 37°C with shaking. 10 μL phosphate-buffered saline (PBS, pH 7.4) was added as untreated control (MOI = 0). Bacterial growth was monitored by measuring optical density at 600 nm at 1-hour intervals for 10 hours using a microplate reader (BioTek, USA). The experiment was performed in triplicates. Prevention of bacterial growth relative to untreated control implied strong lytic ability of the phage.

#### Phage cytotoxicity analysis

RAW264.7 cells were seeded in 96 well plates at a density of 10,000 cells/well in 100 μL of DMEM media and incubated at 37°C with 5% CO_2_ for 24 hours until a confluent monolayer was formed. The cells were then treated with 10 μL of purified phage at a final concentration of 10^5^, 10^6^, and 10^7^ PFU/well, and incubated for 24 hours. 10 μL of commercial *Escherichia coli* O111:B4 LPS (Sigma-Aldrich) was added as a positive control at a final concentration of 20 ng/mL, while wells containing untreated cells served as negative control. Blank control wells containing media only without cells were also included. After incubation, the media was aspirated, and CCK-8 (Cell counting kit-8) solution added to each well, followed by incubation for 2 hours at standard conditions. Cell viability was then assessed by measuring the absorbance of each sample at 450 nm using a microplate reader (BioTek, USA), and subtracting the absorbance of the blank control from each sample absorbance. The percentage of viability was calculated relative to negative control cells (considered 100% viable), using the formula: (Absorbance of treated sample - Absorbance of blank) / (Absorbance of negative control sample - Absorbance of blank) × 100%.

#### Phage internalization studies

A549 and RAW264.7 cells were seeded in 6-well plates (Nunclon Surface, Nunc) at a density of 1 × 10^6^ cells per well, using media supplemented with 2% FBS and antibiotics. After 24 hours incubation, the cell culture medium was discarded, and the cells treated with 1 mL phage diluted in respective media to a final concentration of 1 × 10^9^ PFU/well for 12 hours. After incubation, the cells were thoroughly washed with PBS to remove extracellular phage, and treated with citric acid buffer (40 mM citric acid, 10 mM KCl, 135 mM NaCl, pH 3.0) for 2 minutes to inactivate phage particles adherent on the cell surface.^55^ The cells were washed three times with media to remove residual acid, detached using 0.5% trypsin, and centrifuged three times in PBS at 1500 rpm for 3 minutes to remove extracellular phage. Subsequently, cells were lysed with 0.1% Triton X-100 and the number of intracellular phages after each treatment were determined by spotting tenfold dilutions on a lawn of host bacteria and compared with that of the phage CK01-treated groups.

#### Phage intracellular survival assay

A549 and RAW264.7 cells were seeded in 6-well (Nunclon Surface, Nunc) plates as described above. The cells were treated with 1 mL of phage at a final concentration of 1 × 10^9^ PFU/well for 12 hours. After treatment, the cells were thoroughly washed with PBS to remove extracellular non-adherent phage and incubated for a further 24 hours in fresh media. Following incubation, cells were washed two times with PBS, treated with citric acid buffer, followed by washing and lysis as outlined previously. The phage titer of surviving particles was determined by spotting phage dilutions on host lawn and compared with that of the phage CK01-treated groups.

#### Bacteria-cell coculture and confocal imaging

A549 and RAW264.7 cells were seeded in 35 mm glass-bottom confocal dishes at a density of 1 × 10^6^ cells per dish and allowed to adhere overnight. Bacterial cultures were fluorescently labeled with SYBR™ Green I (1:10,000 dilution in PBS) for 30 minutes at 37°C. Cells were then infected with labeled bacteria at an MOI of 100 in serum-free DMEM and incubated for 4 hours at 37°C under 5% CO_2_.

Following infection, extracellular bacteria were removed by three gentle washes with PBS. Cells were fixed with 4% paraformaldehyde (PFA) for 15 minutes at room temperature, permeabilized with 0.1% Triton X-100 (5 minutes, RT), and blocked with 1% bovine serum albumin (BSA) in PBS for 30 minutes. For membrane visualization, cells were stained with the lipophilic dye DiI (1 μg/mL in PBS) for 15 minutes at 37°C, followed by three PBS washes. Nuclei were counterstained with 4',6-diamidino-2-phenylindole (DAPI, 1 μg/mL) for 15 minutes in the dark. After final PBS washes, samples were imaged using a Leica STELLARIS 8 WILL confocal laser-scanning microscope. Fluorescence signals were captured using sequential scanning to avoid spectral overlaps (SYBR Green I: ex/em 488/520 nm; DiI: ex/em 549/565 nm; DAPI: ex/em 358/461 nm). Image analysis was performed using Leica LAS X and Fiji/ImageJ software.^44^

#### Intracellular killing activity of phage

A549 and RAW264.7 cells were seeded in 24-well plates at a density of 10^5^ cells/well, and incubated for 24 hours (37°C, 5% CO_2_) in media supplemented with 2% FBS. After achieving 80% confluence, the cells were infected with *A. baumannii* bacteria at a bacterium to cell MOI of 10 and incubated at 37°C in 5% CO_2_. One hour post-infection, cells were washed three times with PBS and then incubation with 2 μg/ml polymyxin B (Invitrogen) for 30 minutes at 37°C to remove extracellular bacteria.^23^ Fresh media was added and infected cells incubated for an additional 2 hours. The cells were then washed, treated with 1 mL of respective phage at a final concentration of 10^7^ PFU/well, and incubated for 8 hours. Controls of bacteria-infected cells not treated with phage were included. After incubation, the cells were washed three times with PBS and extracellular phage inactivated as previously described. The cells were then centrifuged to obtain the cell pellet, which was subjected to lysis using 0.1% Triton X-100. The bacteria were then serially diluted and CFU determined.

#### Antimicrobial susceptibility testing

Antimicrobial susceptibility testing was conducted using the broth micro dilution method following the Clinical and Laboratory Standards Institute recommendations. Briefly, Polymyxin B stock was prepared, and two-fold serial dilutions prepared in 96-well plates containing Mueller Hinton broth (MHB) medium. Overnight bacterial cultures were pelleted, washed three times with saline, and reconstituted to the McFarland standard (OD_600_ = 0.1). The standardized bacterial suspension was then diluted 1:20 in MHB, and 10 μL inoculated into the antibiotic dilutions. Negative control wells with MHB medium only (without bacteria) and bacteria without antibiotics were also included. The plate was incubated at 37°C for 24 hours. After incubation, bacterial growth was evaluated by measuring the optical density at 600 nm (OD_600_) using a microplate reader (BioTek, USA). The minimum inhibitory concentration (MIC), defined as the minimum antibiotic concentration to completely inhibit bacterial growth was recorded.

#### RNA preparation and RNA-seq analysis

A549 and RAW264.7 cells were seeded in 6-well plates at a density of 1 × 10^6^ cells per well in media supplemented with 10% FBS and incubated at 37°C, 5% CO_2_ until a confluent monolayer was formed. After incubation, the spent medium was aspirated and replaced with fresh medium containing 2% FBS, adjusted to a final volume of 900 μL per well. A549 and RAW264.7 cells were treated with 100 μL of either PBS (negative control) or diluted phage at 1 × 10^8^ PFU/mL (purified phage diluted to 1 × 10^8^ PFU/mL, leading to a final phage to cell MOI of 10 and 1 × 10^7^ PFU per well). RAW264.7 cells additionally received a background control treatment with commercial *E*. *coli* O111:B4 LPS at 0.02 ng/mL (0.1 EU/mL) to account for potential endotoxin-related immune activation. This allowed subtraction of LPS-induced effects and isolation of phage-specific immunomodulatory responses. Final endotoxin levels in phage preparations at 1 × 10^8^ PFU/mL used for RAW264.7 cells were 0.995, 0.96, and 0.980 EU/mL for phage CK02, CK12, and CK21, respectively (Table S1). RAW264.7 cells incubated with each phage for 6 and 24 hours were collected for RNA-seq analysis. Final endotoxin levels in phage preparations at 1 × 10^8^ PFU/mL applied to A549 cells were 15.1, 9, 15.2, 19, 23, and 13.3 EU/mL for phage CK01, CK02, CK10, CK11, CK12, and CK21, respectively (Table S1). A549 cells incubated with each phage for 12 hours were collected for RNA-seq analysis.

After incubation, cells were thoroughly washed with sterile PBS to remove extracellular phage. Total RNA was prepared using TRIzol^56^ and the samples stored at -80°C prior to RNA extraction. RNA extraction and RNA-seq analysis of A549 cells and RAW264.7 cells were performed by the Personalbio Technology Company (Shanghai, China) and the Beijing Novogene Technology Co., Ltd. (Beijing, China), respectively. Briefly, after the total RNA extraction, quality inspection, and DNase I (TaKaRa Bio) treatment, polyA-enriched mRNA was isolated using oligo dT magnetic beads and fragmented into short fragments at high temperatures using divalent cations. Then cDNA was synthesized by reverse transcription followed by end repair, adenylation of the 3’ end of cDNA fragments. Adapter ligation was performed using pair-end adapters (Illumina). cDNA fragments were then purified by gel electrophoresis and enriched by PCR amplification to create the final cDNA libraries. The libraries were quality checked using Agilent 2100 Bioanalyzer and subjected to paired-end (PE) sequencing on Illumina sequencing platform.

#### Transcriptome assembly

Image files were generated by the sequencer and transformed into nucleotide sequences which were saved in FASTQ format (Raw data). Quality control was performed to detect the qualification of the data. The filtration of low-quality reads was performed using the Cutadapt software to remove the 3’ end of the linker and reads with an average quality score lower than Q20.^49^ After filtering, the clean reads were mapped to the reference sequences using TopHat2’s^24^ upgraded version of HISAT2^25^ software to align the filtered reads to the reference human (GRCh37) and mouse (GRCm38) genome.

#### Transcriptional profiling analysis

HTSeq was used to analyze expression level of each gene.^51^ To ensure comparability of gene expression levels across different genes and samples, fragments per kilo base per million mapped reads (FPKM) were used for normalization. In this experiment, genes with FPKM > 1 were considered as expressed. DESeq was used for differential analysis of gene expressions. The conditions for screening differentially expressed genes included: expression difference log_2_Fold change > 1, and significance *p*-value < 0.05.^57^

#### Functional enrichment analysis

Functional enrichment analysis was performed on differentially expressed genes associated with treatment responses, to interpret their biological functions. Gene Ontology (GO) functional classifications were defined by the topGO software, and the enriched gene functional categories were further classified by GO analysis, with *p-value* < 0.05. The Kyoto Encyclopedia of Genes and Genomes (KEGG) pathway database was accessed using the clusterprofiler software,^50^ with a corrected *p-value* < 0.05.

#### Phage genome extraction, sequencing, and annotation

The genomic DNA of *A. baumannii* phage was extracted using the phenol: chloroform method. Phage lysates were initially concentrated using 1 M NaCl and 10% PEG and purified by ultracentrifugation. The purified lysate was treated with DNase I and RNase at 37°C for 1 hour to eliminate contaminating host nucleic acids. The DNase and RNase were inactivated using 0.5 M EDTA and heated at 72°C for 10 minutes. Subsequently, the phage was lysed using proteinase K and subjected to three rounds of phenol-chloroform-isopropyl alcohol (24:1:25, vol/vol) treatment to remove proteins and lipids. The DNA was then precipitated with 100% ethanol and 0.3 M sodium acetate (pH 5.2) at 20°C overnight, centrifuged at 12,000 ×g for 30 minutes at 4°C, and the DNA pellet resuspended in TE buffer. The purity, concentration and quality of the extracted DNA were confirmed using a NanoDrop 2000c spectrophotometer (Thermo Scientific, USA) and 1% agarose gel electrophoresis, ensuring suitability for downstream applications.

The complete genomes were sequenced by Shanghai Personalbio Tech Company, China, using the Illumina Novaseq platform. The process involved preparing sequencing libraries from the high-quality DNA, followed by high-throughput paired-end sequencing. The raw reads were processed to filter out low-quality sequences, retaining only high-quality reads for assembly. Genome assembly was performed using SPAdes, producing contigs and scaffolds for subsequent analysis. Coding genes were predicted using the GeneMarkS software (Version 4.28).^46^ tRNAs were predicted using tRNAscan-SE software (Version 1.3.1).^58^ CRISPR arrays were identified using CRISPRdigger (Version 1.0).^59^

Functional annotation of coding genes was performed using multiple databases, including GO, KEGG, COG/KOG, NR, Pfam, CAZy, TCDB, Swiss-Prot, and Prokka.^45^ Additional functional prediction was achieved using NCBI BLASTp and BLASTx against the nr protein database, blast conserved domains.^47^ Lifestyle prediction was performed using PhageScope.^48^ Antibiotic-resistance genes were predicted using the Virulence Factor Database (VFDB, http://www.mgc.ac.cn/VFs/main.htm) and the Comprehensive Antibiotic Resistance Database (CARD, https://card.mcmaster.ca/analyze/rgi).^60^ For comparative genome analysis, the phage whole genomes were used as the query for computing genome-wide sequence similarities using tBLASTx against other DsDNA viral genome sequences on VipTree V1.9 (https://www.genome.jp/VIPtree/)^21^ accessed on March 16^th^ 2025. VICTOR web service (https://victor.dsmz.de) was employed for genome-based phylogeny classification where all pairwise comparisons were conducted using the Genome-BLAST Distance Phylogeny (GBDP) method.^22^ Intergenomic nucleotide sequence similarity among the *A. baumannii* phage using Virus Intergenomic Distance Calculator (VIRIDIC). The genomic similarity threshold was set at 70% for genus and 95% for species.^61^

### Quantification and statistical analysis

Statistical analyses were conducted using Prism 9.3 software (GraphPad Inc.). Phage internalization, intracellular survival, and intracellular antibacterial activity data was analyzed by one-way ANOVA. Data are presented as mean values or mean ± SD of three biological replicates, unless otherwise specified. All of the statistical details of experiments can be found in the figure legends and methods section of the manuscript.

## Supplemental information

Document S1. Figures S1–S6 and Tables S1–S3, S9, and S10

Table S4. DEGs in A549 cells treated with phage CK01, CK02, CK10, CK11, CK12, and CK21 for 12 h, related to Figure 3A

Table S5. DEGs in RAW264.7 cells treated with phage CK02 for 6 and 24 h, related to Figure 3B

Table S6. DEGs in RAW264.7 cells treated with phage CK12 for 6 and 24 h, related to Figure 3B

Table S7. DEGs in RAW264.7 cells treated with phage CK21 for 6 and 24 h, related to Figure 3B

Table S8. DEGs in RAW264.7 cells treated with LPS for 6 and 24 h, related to Figure 3B
