# Supplementary material for: Transcriptomic analysis of phage-mammalian cell interaction reveals diverse phage immunobarcodes
Source: iScience. 2025 Sep 5;28(10):113513. doi: 10.1016/j.isci.2025.113513 (PMC12495068; doi:10.1016/j.isci.2025.113513)
Supplement: Document S1. Figures S1–S6 and Tables S1–S3, S9, and S10 [file mmc1.pdf]

iScience, Volume *28*

## Supplemental information

# Transcriptomic analysis of phage-mammalian cell interaction reveals diverse phage immunobarcodes

**Caroline Munini Muema, Belinda Kibii, Mingyue Zhong, Xinfeng Li, Paulina Miernikiewicz, Heng Xue, Yiyao Wang, Raphael Nyaruaba, Krystyna Dąbrowska, Hongping Wei, and Hang Yang**

## Supplementary information

## Figure S1. Genome maps of the *A. baumannii* phage related to Figure 1

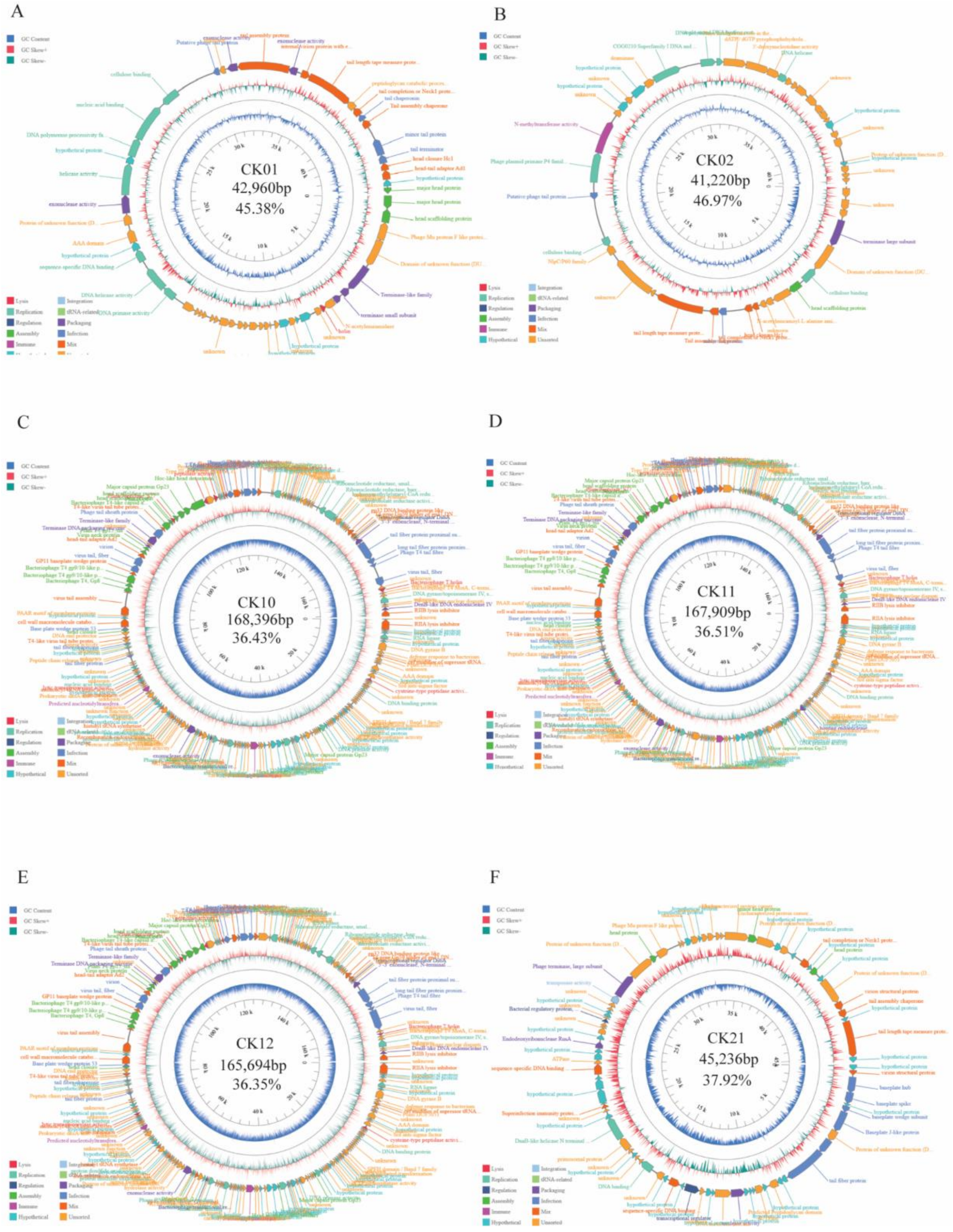


**Figure S1. Genome maps of *A. baumannii* phage used in this study.** Genome circos maps showing the genomic organization of *A. baumannii* phage. ORFs with putative annotations are represented by specific colours as per their functional categories marked using arrows. The legends on the left of each map show the different functional categories and their colours.

**Figure S2. Taxonomic classification of *A. baumannii* phage related to Figure 1**

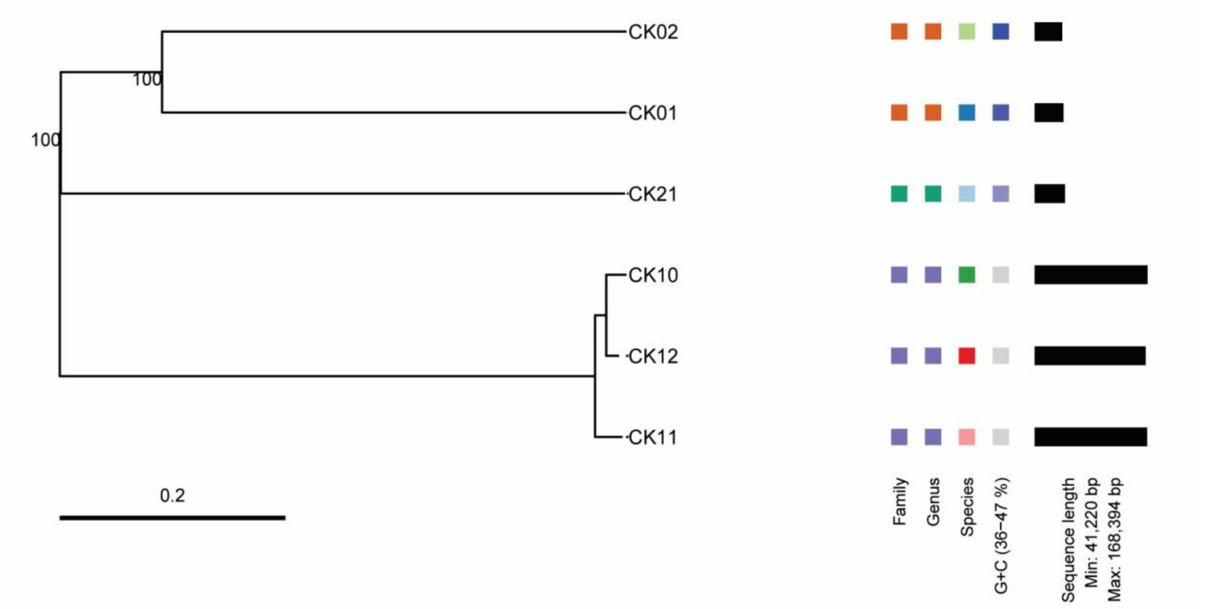


**Figure S2. Phylogenetic analysis of *A. baumannii* phage used in this study.** Genome-BLAST Distance Phylogeny (GBDP) phylogram of the *A. baumannii* phage based on the whole genome sequences inferred using formula D0. The number at each node is the GBDP pseudo-bootstrap support value from 100 replications. Phage genera are assigned according to the official ICTV classification and represented with different colour frames. The GC content and sequence length of each phage genome is indicated on the right.

## Figure S3. Phage concentration and endotoxin removal process, related to Figure 1

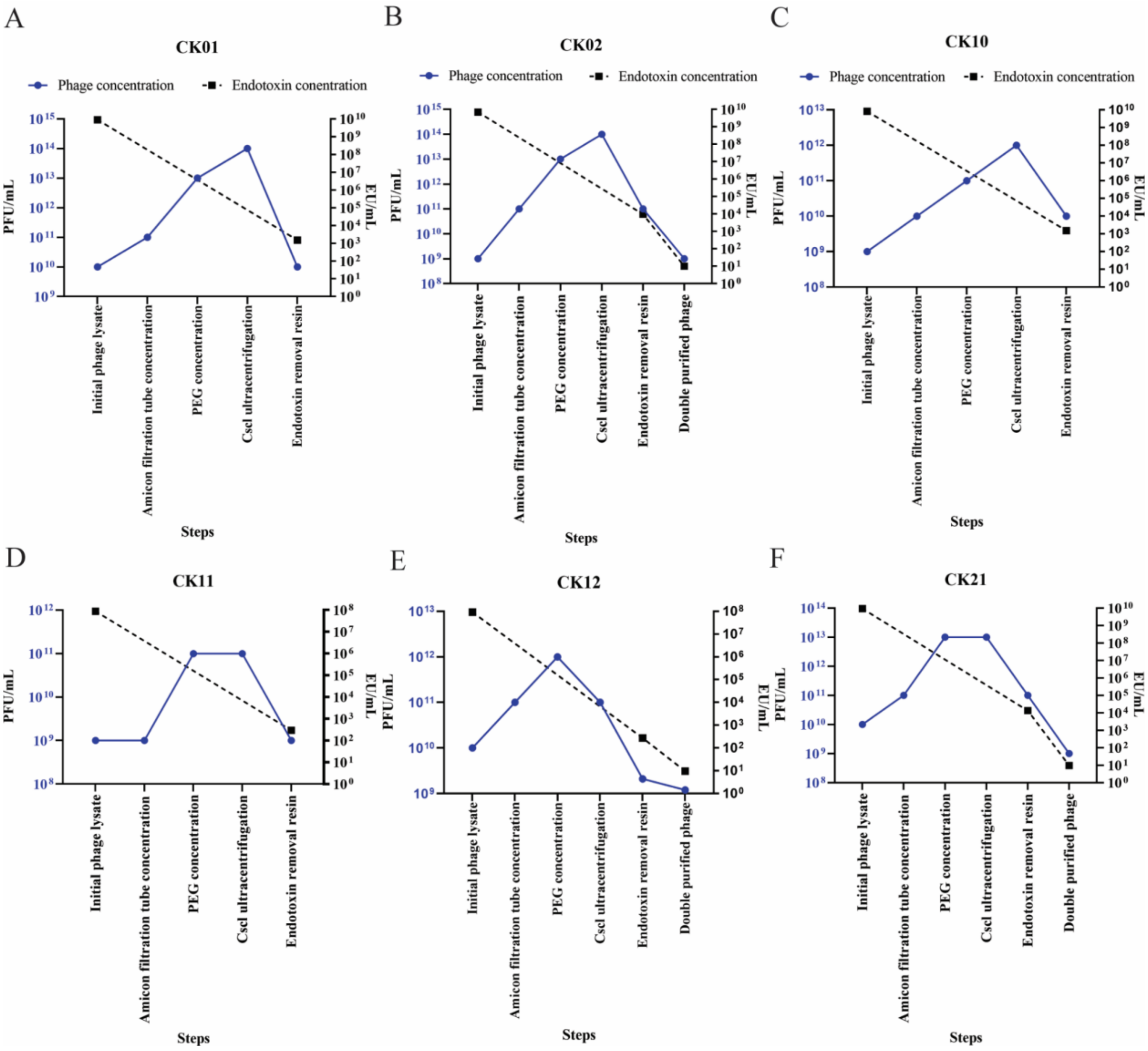


**Figure S3. Phage safety analysis**. (a-f) Stepwise concentration of *A. baumannii* phage CK01, CK02, CK10, CK11, CK12, and CK21, and endotoxin levels. PFUs per mL (right *y*-axis; closed circles) and endotoxin units (EUs) (left *x*-axis, closed squares) after phage lysate filtration, Amicon tube concentration, PEG concentration, CsCl density gradient ultracentrifugation and finally endotoxin removal resin. Graphs represent the mean of three technical replicates.

**Figure S4. Phage do not elicit cytotoxic effects in RAW264.7 cells, related to Figure 1**

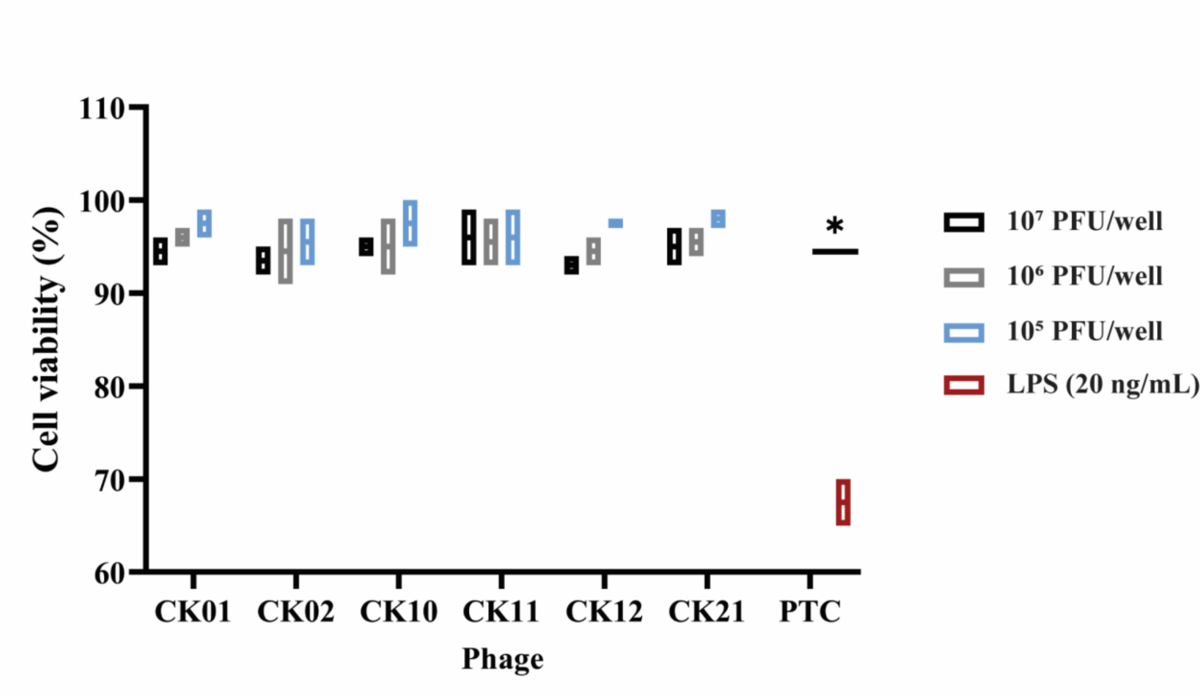


**Figure S4. Effect of phage treatment on cell viability.** The relative viability of murine macrophage RAW264.7 cells after 24-hour exposure to various concentrations of different phage and LPS (positive control) compared to untreated cells. Data are presented as mean ± standard deviation (SD) from three independent experiments. A two-sample t-test comparing phage treatment (mean of the relative %viability of each phage at $10^8$ PFU/well) and LPS control indicated a statistically significant difference ($p = 0.038$, *: $p \leq 0.05$).

**Figure S5. Confocal microscopy images showing intracellular *A. baumannii* related to Figure 2C**

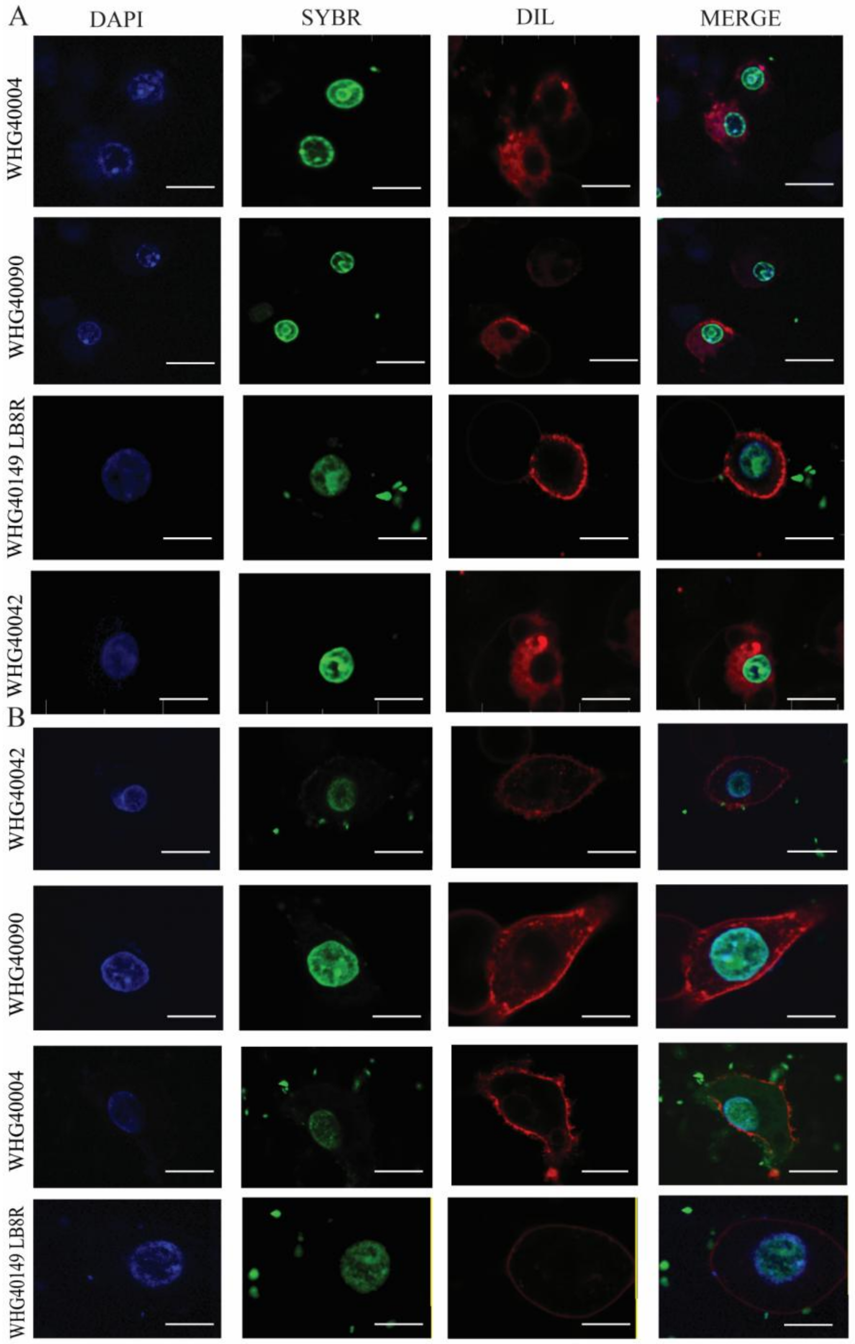


**Figure S5. Laser scanning electron microscopy showing internalized *A. baumannii* strains in (A) RAW2654.7 and (B) A549 cells.** DAPI: cell nucleus (blue), SYBR Green: Bacteria (green), and Dil: Cell membrane (red). Scale bars, 5 µm.

**Figure S6. Pearson correlation between different treatment samples in A549 and RAW264.7 cells, related to Figure 3A and 3B**

A

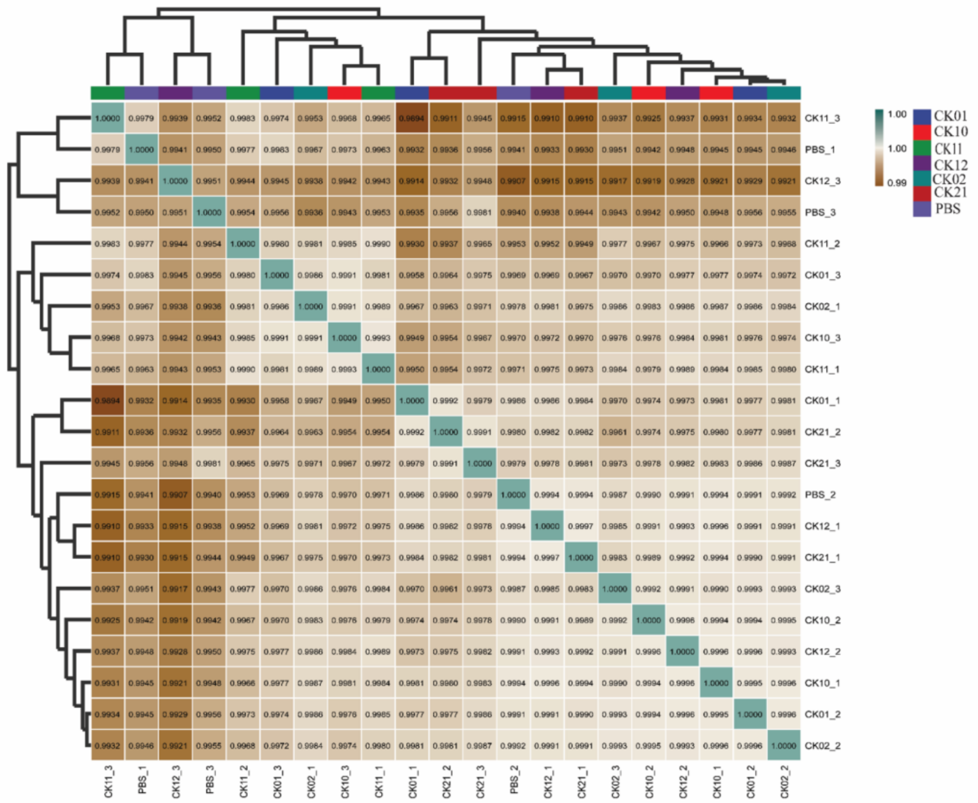


B

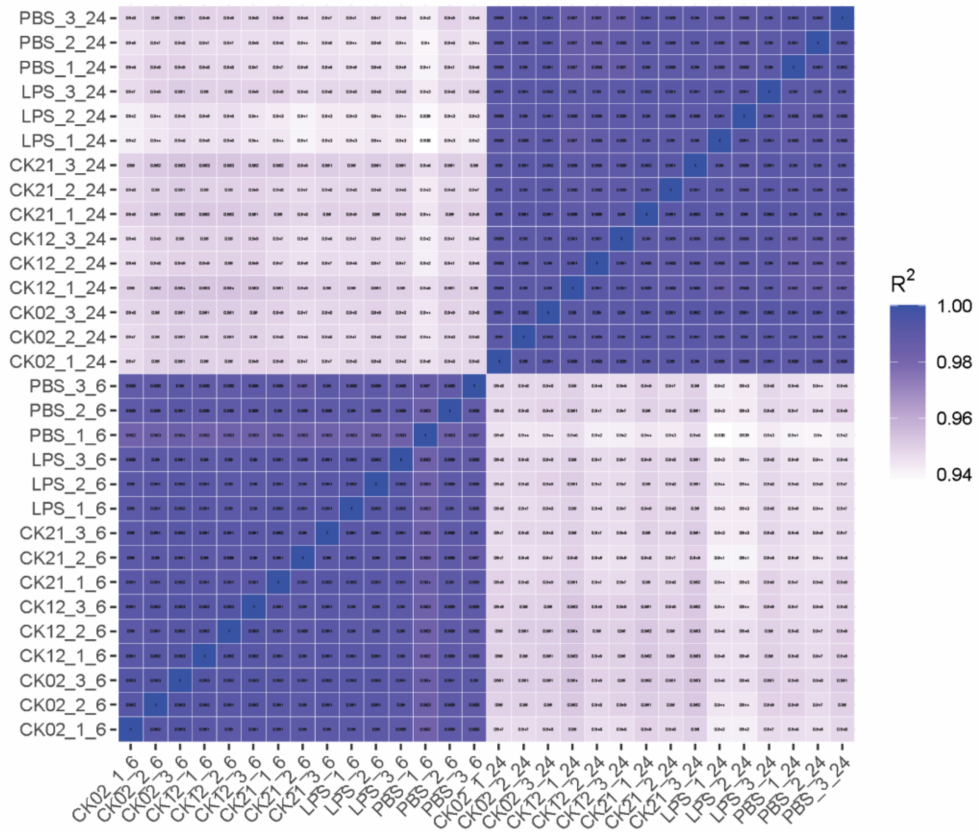

**Figure S6. Sample correlation in (A) A549 cells and (B) RAW264.7 cells transcriptome.** Heat maps showing the correlation between phage and control treatment samples based on fpkm values of DEGs in each sample. The scale represents the $R^2$ correlation coefficient value. The horizontal and vertical coordinates in the figure are the squares of the correlation coefficients of each sample.

## Table S1. Phage and bacteria information, related to Figure 1

| **Phage** | **vB_AbaS_CK01** | **vB_AbaS_CK02** | **vB_AbaM_CK10** | **vB_AbaM_CK11** | **vB_AbaM_CK12** | **vB_AbaP_CK21** |
|---|---|---|---|---|---|---|
| **Phage name abbreviation** | **CK01** | **CK02** | **CK10** | **CK11** | **CK12** | **CK21** |
| **Host** | *A. baumannii* strain WHG40042 | *A. baumannii* strain WHG40042 | *A. baumannii* strain WHG40090 | *A. baumannii* strain WHG40149 LB8R | *A. baumannii* strain WHG40149 LB8R | *A. baumannii* strain WHG40004 |
| **Host susceptibility to polymyxin B (MIC, µg/ml)** | 1.25 | 1.25 | 1.25 | 1.25 | 1.25 | 1.25 |
| **Source** | Sewage | Wastewater | Wastewater | Wastewater | Pond water | Wastewater |
| **Place of phage isolation** | Huazhong agricultural university | Huazhong agricultural university | Tongji hospital | Jiangxia hospital | Pond | Xiehe hospital |
| **Morphology** | Siphovirus | Siphovirus | Myovirus | Myovirus | Myovirus | Podovirus |
| **Head diameter (nm)** | 78.73 | 52.19 | 111.95 | 79.07 | 145.61 | 103.46 |
| **Tail length (nm)** | 269 | 211 | 174.61 | 164.71 | 284.8 | 22.6 |
| **Tail width (nm)** | 12.86 | 13.48 | 26.71 | 16.51 | 32.88 | 11.68 |
| **Endotoxin concentration in phage preps before A549 cells treatment** | 15.1 EU/mL in $1\times10^8$ PFU/mL | 9 EU/mL in $1\times10^8$ PFU/mL | 15.2 EU/mL in $1\times10^8$ PFU/mL | 29 EU/mL in $1\times10^8$ PFU/mL | 23 EU/mL in $1\times10^8$ PFU/mL | 13.3 EU/mL in $1\times10^8$ PFU/mL |
| **Endotoxin concentration in phage preps after a second round of purification before RAW264.7 cells treatment** | - | 0.995 EU/mL in $1\times10^8$ PFU/mL | - | - | 0.961 EU/mL in $1\times10^8$ PFU/mL | 0.980 EU/mL in $1\times10^8$ PFU/mL |
| **Genomic features** | | | | | | |
| **Nucleic acid** | dsDNA | dsDNA | dsDNA | dsDNA | dsDNA | dsDNA |
| **Genome size (bp)** | 42,960 | 41,220 | 168,396 | 167,909 | 165,694 | 45,236 |
| **GC content (%)** | 45.38 | 46.97 | 36.43 | 36.51 | 36.35 | 37.92 |
| **Number of features** | 58 | 49 | 250 | 250 | 251 | 87 |
| **Class** | Caudoviricetes | Caudoviricetes | Caudoviricetes | Caudoviricetes | Caudoviricetes | Caudoviricetes |
| **Phage family** | Unclassified | Unclassified | Straboviridae | Straboviridae | Straboviridae | Unclassified |
| **Phage genus (based on Phagenomics)** | Lokivirus | Unclassified | Hadassahvirus | Hadassahvirus | Hadassahvirus | Obolenskvirus |
| **Lifestyle (based on PhageScope)** | Virulent | Virulent | Virulent | Virulent | Virulent | Virulent |
| **Accession number** | C_AA107471.1 | C_AA107472.1 | C_AA107473.1 | C_AA107474.1 | C_AA107475.1 | C_AA107476.1 |

'- 'experiment not performed.

## Table S2. Summary of transcriptome profile of A549 cells, related to Figure 3A

| Sample | Clean Reads | Total Mapped | Multiple Mapped | Uniquely Mapped | Map Events | Mapped to Gene | Mapped to InterGene | Mapped to Exon |
|---|---|---|---|---|---|---|---|---|
| CK01_1 | 45082334 | 43529421 (96.56%) | 1791798 (4.12%) | 41737623 (95.88%) | 41737623 | 41255997 (98.85%) | 481626 (1.15%) | 38442878 (93.18%) |
| CK01_2 | 50939458 | 48747849 (95.70%) | 1787136 (3.67%) | 46960713 (96.33%) | 46960713 | 46467007 (98.95%) | 493706 (1.05%) | 43854036 (94.38%) |
| CK01_3 | 43228366 | 41524976 (96.06%) | 1452310 (3.50%) | 40072666 (96.50%) | 40072666 | 39698608 (99.07%) | 374058 (0.93%) | 37726548 (95.03%) |
| CK02_1 | 48799130 | 46796660 (95.90%) | 1695370 (3.62%) | 45101290 (96.38%) | 45101290 | 44651205 (99.00%) | 450085 (1.00%) | 42122595 (94.34%) |
| CK02_2 | 45095760 | 43227770 (95.86%) | 1523986 (3.53%) | 41703784 (96.47%) | 41703784 | 41290434 (99.01%) | 413350 (0.99%) | 39005635 (94.47%) |
| CK02_3 | 41644924 | 39865682 (95.73%) | 1379540 (3.46%) | 38486142 (96.54%) | 38486142 | 38105686 (99.01%) | 380456 (0.99%) | 35826336 (94.02%) |
| CK10_1 | 42351518 | 40483200 (95.59%) | 1507812 (3.72%) | 38975388 (96.28%) | 38975388 | 38562609 (98.94%) | 412779 (1.06%) | 36265470 (94.04%) |
| CK10_2 | 40958598 | 39016899 (95.26%) | 1266488 (3.25%) | 37750411 (96.75%) | 37750411 | 37363458 (98.97%) | 386953 (1.03%) | 34995513 (93.66%) |
| CK10_3 | 44612172 | 42939525 (96.25%) | 1511622 (3.52%) | 41427903 (96.48%) | 41427903 | 41027636 (99.03%) | 400267 (0.97%) | 38682379 (94.28%) |
| CK11_1 | 42560162 | 41010589 (96.36%) | 1814104 (4.42%) | 39196485 (95.58%) | 39196485 | 38750213 (98.86%) | 446272 (1.14%) | 36356079 (93.82%) |
| CK11_2 | 42015846 | 40345351 (96.02%) | 1523918 (3.78%) | 38821433 (96.22%) | 38821433 | 38453996 (99.05%) | 367437 (0.95%) | 36512940 (94.95%) |
| CK11_3 | 41333808 | 39894803 (96.52%) | 1543690 (3.87%) | 38351113 (96.13%) | 38351113 | 37992438 (99.06%) | 358675 (0.94%) | 36146655 (95.14%) |
| CK12_1 | 52015366 | 49413489 (95.00%) | 1650855 (3.34%) | 47762634 (96.66%) | 47762634 | 47251094 (98.93%) | 511540 (1.07%) | 44301624 (93.76%) |
| CK12_2 | 51169866 | 48844134 (95.45%) | 1623096 (3.32%) | 47221038 (96.68%) | 47221038 | 46748683 (99.00%) | 472355 (1.00%) | 43955909 (94.03%) |
| CK12_3 | 47268574 | 45313562 (95.86%) | 1614737 (3.56%) | 43698825 (96.44%) | 43698825 | 43270730 (99.02%) | 428095 (0.98%) | 40836170 (94.37%) |
| CK21_1 | 40109670 | 38468721 (95.91%) | 1273708 (3.31%) | 37195013 (96.69%) | 37195013 | 36823870 (99.00%) | 371143 (1.00%) | 34677728 (94.17%) |
| CK21_2 | 46644630 | 45025180 (96.53%) | 1602187 (3.56%) | 43422993 (96.44%) | 43422993 | 42955758 (98.92%) | 467235 (1.08%) | 40393671 (94.04%) |
| CK21_3 | 45511964 | 43967595 (96.61%) | 1594002 (3.63%) | 42373593 (96.37%) | 42373593 | 41952896 (99.01%) | 420697 (0.99%) | 39872371 (95.04%) |
| PBS_1 | 41711462 | 40427182 (96.92%) | 1448610 (3.58%) | 38978572 (96.42%) | 38978572 | 38624742 (99.09%) | 353830 (0.91%) | 36448260 (94.37%) |
| PBS_2 | 49556798 | 47684242 (96.22%) | 1652954 (3.47%) | 46031288 (96.53%) | 46031288 | 45558781 (98.97%) | 472507 (1.03%) | 42502488 (93.29%) |
| PBS_3 | 41319542 | 39452451 (95.48%) | 1389421 (3.52%) | 38063030 (96.48%) | 38063030 | 37730197 (99.13%) | 332833 (0.87%) | 36086491 (95.64%) |

## Table S3. Summary of transcriptome profile of RAW264.7 cells, related to Figure 3B

| Sample | Total_reads | Total_map | Unique_map | Multi_map | Read1_map | Read2_map | Positive_map | Negative_map | Splice_map | Unsplice_map | Proper_map |
|---|---|---|---|---|---|---|---|---|---|---|---|
| CK02_1_6 | 44067408 | 39043730 (88.6%) | 35996400 (81.68%) | 3047330 (6.92%) | 18041267 (40.94%) | 17955133 (40.74%) | 17995373 (40.84%) | 18001027 (40.85%) | 17018811 (38.62%) | 18977589 (43.06%) | 33951992 (77.05%) |
| CK02_2_6 | 49666324 | 43963550 (88.52%) | 40505042 (81.55%) | 3458508 (6.96%) | 20303789 (40.88%) | 20201253 (40.67%) | 20246260 (40.76%) | 20258782 (40.79%) | 19179216 (38.62%) | 21325826 (42.94%) | 38202656 (76.92%) |
| CK02_3_6 | 69529578 | 62128064 (89.35%) | 57271783 (82.37%) | 4856281 (6.98%) | 28694817 (41.27%) | 28576966 (41.1%) | 28632792 (41.18%) | 28638991 (41.19%) | 27073754 (38.94%) | 30198029 (43.43%) | 54285766 (78.08%) |
| CK12_1_6 | 45926842 | 40685095 (88.59%) | 37520693 (81.7%) | 3164402 (6.89%) | 18811550 (40.96%) | 18709143 (40.74%) | 18759292 (40.85%) | 18761401 (40.85%) | 17537278 (38.19%) | 19983415 (43.51%) | 35296076 (76.85%) |
| CK12_2_6 | 55356676 | 48912415 (88.36%) | 45025195 (81.34%) | 3887220 (7.02%) | 22572801 (40.78%) | 22452394 (40.56%) | 22507429 (40.66%) | 22517766 (40.68%) | 21048489 (38.02%) | 23976706 (43.31%) | 42220918 (76.27%) |
| CK12_3_6 | 55743096 | 49372158 (88.57%) | 45494047 (81.61%) | 3878111 (6.96%) | 22807585 (40.92%) | 22686462 (40.7%) | 22743601 (40.8%) | 22750446 (40.81%) | 21329243 (38.26%) | 24164804 (43.35%) | 42788344 (76.76%) |
| CK21_1_6 | 52722388 | 46886685 (88.93%) | 43239800 (82.01%) | 3646885 (6.92%) | 21676443 (41.11%) | 21563357 (40.9%) | 21613367 (40.99%) | 21626433 (41.02%) | 20162821 (38.24%) | 23076979 (43.77%) | 40904320 (77.58%) |
| CK21_2_6 | 39486196 | 35471084 (89.83%) | 32737806 (82.91%) | 2733278 (6.92%) | 16409933 (41.56%) | 16327873 (41.35%) | 16366545 (41.45%) | 16371261 (41.46%) | 15471907 (39.18%) | 17265899 (43.73%) | 31273958 (79.2%) |
| CK21_3_6 | 52044592 | 45939935 (88.27%) | 42209995 (81.1%) | 3729940 (7.17%) | 21158691 (40.65%) | 21051304 (40.45%) | 21098329 (40.54%) | 21111666 (40.56%) | 19657206 (37.77%) | 22552789 (43.33%) | 39482778 (75.86%) |
| LPS_1_6 | 51512098 | 45989616 (89.28%) | 42420890 (82.35%) | 3568726 (6.93%) | 21263053 (41.28%) | 21157837(41.07%) | 21209045 (41.17%) | 21211845 (41.18%) | 20212323 (39.24%) | 22208567 (43.11%) | 40295824 (78.23%) |
| LPS_2_6 | 47265670 | 42085418 (89.04%) | 38777708 (82.04%) | 3307710 (7.0%) | 19442588 (41.13%) | 19335120 (40.91%) | 19387034 (41.02%) | 19390674 (41.02%) | 18398168 (38.93%) | 20379540 (43.12%) | 36617978 (77.47%) |
| LPS_3_6 | 42643714 | 37947820 (88.99%) | 34964724 (81.99%) | 2983096 (7.0%) | 17529225 (41.11%) | 17435499 (40.89%) | 17478563 (40.99%) | 17486161 (41.01%) | 16539031 (38.78%) | 18425693 (43.21%) | 32983288 (77.35%) |
| PBS_1_6 | 54283022 | 50786326 (93.56%) | 46664456 (85.97%) | 4121870 (7.59%) | 23373401 (43.06%) | 23291055 (42.91%) | 23326546 (42.97%) | 23337910 (42.99%) | 22965014 (42.31%) | 23699442 (43.66%) | 45773394 (84.32%) |
| PBS_2_6 | 39413170 | 34987966 (88.77%) | 32255360 (81.84%) | 2732606 (6.93%) | 16169369 (41.03%) | 16085991 (40.81%) | 16125341 (40.91%) | 16130019 (40.93%) | 14937564 (37.9%) | 17317796 (43.94%) | 30325806 (76.94%) |
| PBS_3_6 | 44639866 | 40110757 (89.85%) | 36873146(82.6%) | 3237611 (7.25%) | 18480047 (41.4%) | 18393099 (41.2%) | 18430140(41.29%) | 18443006 (41.32%) | 17557148 (39.33%) | 19315998 (43.27%) | 34569646 (77.44%) |
| CK02_1_24 | 46629852 | 40793559 (87.48%) | 37625169 (80.69%) | 3168390 (6.79%) | 18856653 (40.44%) | 18768516 (40.25%) | 18802740(40.32%) | 18822429 (40.37%) | 17042002 (36.55%) | 20583167 (44.14%) | 36174336 (77.58%) |
| CK02_2_24 | 47848244 | 41807538 (87.38%) | 38610947 (80.69%) | 3196591 (6.68%) | 19356085 (40.45%) | 19254862 (40.24%) | 19302204 (40.34%) | 19308743 (40.35%) | 17147563 (35.84%) | 21463384 (44.86%) | 36268548 (75.8%) |
| CK02_3_24 | 54780510 | 48142419 (87.88%) | 44498431 (81.23%) | 3643988 (6.65%) | 22303055 (40.71%) | 22195376 (40.52%) | 22241994 (40.6%) | 22256437 (40.63%) | 20144770 (36.77%) | 24353661 (44.46%) | 42060862 (76.78%) |
| CK12_1_24 | 47374746 | 41200303 (86.97%) | 38067832 (80.35%) | 3132471 (6.61%) | 19080299 (40.28%) | 18987533 (40.08%) | 19034044 (40.18%) | 19033788 (40.18%) | 17295775 (36.51%) | 20772057 (43.85%) | 35950198 (75.88%) |
| CK12_2_24 | 45253752 | 38975663 (86.13%) | 36012735 (79.58%) | 2962928 (6.55%) | 18048888 (39.88%) | 17963847 (39.7%) | 18003657 (39.78%) | 18009078 (39.8%) | 16026124 (35.41%) | 19986611 (44.17%) | 33732580 (74.54%) |
| CK12_3_24 | 51752158 | 44500587 (85.99%) | 41105995 (79.43%) | 3394592 (6.56%) | 20605195 (39.82%) | 20500800 (39.61%) | 20548778 (39.71%) | 20557217 (39.72%) | 18203757 (35.17%) | 22902238 (44.25%) | 38442738 (74.28%) |
| CK21_1_24 | 55148466 | 47849785 (86.77%) | 44221552 (80.19%) | 3628233 (6.58%) | 22170021 (40.2%) | 22051531 (39.99%) | 22107271 (40.09%) | 22114281 (40.1%) | 19478258 (35.32%) | 24743294 (44.87%) | 41092850 (74.51%) |
| CK21_2_24 | 38639624 | 33738223 (87.32%) | 31226610(80.81%) | 2511613(6.5%) | 15652886(40.51%) | 15573724(40.31%) | 15611519(40.4%) | 15615091(40.41%) | 13963001(36.14%) | 17263609(44.68%) | 29427058(76.16%) |
| CK21_3_24 | 48145020 | 41879312 (86.99%) | 38719178(80.42%) | 3160134(6.56%) | 19410582(40.32%) | 19308596(40.11%) | 19356857(40.21%) | 19362321(40.22%) | 17292112(35.92%) | 21427066(44.51%) | 36299376(75.4%) |
| LPS_1_24 | 47501156 | 41562534 (87.5%) | 38527442(81.11%) | 3035092(6.39%) | 19308878(40.65%) | 19218564(40.46%) | 19260101(40.55%) | 19267341(40.56%) | 17170532(36.15%) | 21356910(44.96%) | 36113040(76.03%) |

| | | | | | | | | | | | |
|---|---|---|---|---|---|---|---|---|---|---|---|
| LPS_2_24 | 45755116 | 40013190 (87.45%) | 37009442(80.89%) | 3003748(6.56%) | 18553535(40.55%) | 18455907(40.34%) | 18499748(40.43%) | 18509694(40.45%) | 16562019(36.2%) | 20447423(44.69%) | 34735870(75.92%) |
| LPS_3_24 | 51888922 | 45353626 (87.41%) | 41928276(80.8%) | 3425350(6.6%) | 21015791(40.5%) | 20912485(40.3%) | 20961840(40.4%) | 20966436(40.41%) | 18799099(36.23%) | 23129177(44.57%) | 39482156(76.09%) |
| PBS_1_24 | 49358962 | 43097299 (87.31%) | 39833302(80.7%) | 3263997(6.61%) | 19967888(40.45%) | 19865414(40.25%) | 19912480(40.34%) | 19920822(40.36%) | 17579555(35.62%) | 22253747(45.09%) | 37667528(76.31%) |
| PBS_2_24 | 47796662 | 41777874 (87.41%) | 38682240(80.93%) | 3095634(6.48%) | 19387723(40.56%) | 19294517(40.37%) | 19337197(40.46%) | 19345043(40.47%) | 17051032(35.67%) | 21631208(45.26%) | 36442792(76.25%) |
| PBS_3_24 | 54141198 | 47298599 (87.36%) | 43730415(80.77%) | 3568184(6.59%) | 21920310(40.49%) | 21810105(40.28%) | 21860839(40.38%) | 21869576(40.39%) | 19281660(35.61%) | 24448755(45.16%) | 41247032(76.18%) |

## Tables S4, S5, S6, S7, and S8 are available as excel files

**Table S9. Commonly expressed genes in A549 cells after challenge with phage, related to Figure 4A.**

| Gene_id | CK01_vs_PBS _log2fc | CK02_vs_PBS _log2fc | CK10_vsPBS _log2fc | CK11_vs_PBS _log2fc | CK12_vs_PBS _log2fc | CK21_vs_PBS _log2fc |
|---|---|---|---|---|---|---|
| PER1 | 1.939953 | 1.849261 | 2.045994 | 2.058366 | 2.203933 | 1.978742 |
| IL11 | -1.5392 | -2.06496 | -1.93127 | -2.09769 | -1.77073 | -1.43334 |
| CYP1A1 | 2.986072 | 2.750001 | 2.582638 | 2.973143 | 2.375822 | 2.871894 |
| TIPARP | 1.217712 | 1.310456 | 1.217064 | 1.155387 | 1.413565 | 1.358852 |
| FKBP5 | 1.166801 | 1.247177 | 1.17287 | 1.040446 | 1.210235 | 1.156965 |
| KLF9 | 1.634837 | 1.898553 | 1.781946 | 1.48044 | 1.619227 | 1.488758 |
| AHRR | 1.304264 | 1.529701 | 1.413412 | 1.64693 | 1.299257 | 1.379588 |
| CYP1B1 | 1.312391 | 1.279323 | 1.29101 | 1.077953 | 1.421961 | 1.404442 |
| SOX9 | -1.0269 | -1.80164 | -1.46606 | -1.64462 | -1.4747 | -1.30774 |
| ID4 | -1.03869 | -1.53141 | -1.00245 | -1.40246 | -1.34067 | -1.33005 |
| MYOCD | -1.39197 | -1.70875 | -1.44788 | -2.06311 | -1.60883 | -1.23687 |
| BBS1 | 1.666257 | 1.74467 | 1.716409 | 1.654986 | 1.815365 | 1.7549 |
| MCF2L | 1.219137 | 1.477728 | 1.090152 | 1.514688 | 1.004389 | 1.167731 |

**Table S10: Commonly expressed genes in RAW 264.7 cells after challenge with phage, related to Figure 4B.**

| Gene_id | CK02 vs PBS_24 | CK12 vs PBS_24 | CK21 vs PBS_24 |
|---|---|---|---|
| 4933426B08Rik | 1.37889 | 1.30606 | 1.15421 |
| Gm50270 | -3.45773 | -2.33843 | -2.65855 |
| AC122821.1 | -4.82813 | -4.75494 | -4.72601 |
| Tnxb | -1.45661 | -2.3832 | -1.40213 |
| F2 | 4.36387 | 5.08867 | 5.03714 |
| Gm43164 | -2.12766 | -2.24684 | -3.96003 |
| Gm38346 | -1.0915 | -1.45678 | -1.11569 |
